# Structural identification of pyridinopyrone compounds with anti-neuroinflammatory activity from streptomyces sulphureus DSM 40104

**DOI:** 10.3389/fmicb.2023.1205118

**Published:** 2023-06-01

**Authors:** Juan Hu, Zi-Xuan Wang, Pei-Meng Li, Pei-Yuan Qian, Ling-Li Liu

**Affiliations:** ^1^Shaanxi Key Laboratory of Natural Products & Chemical Biology, College of Chemistry & Pharmacy, Northwest A&F University, Yangling, Shaanxi, China; ^2^Southern Marine Science and Engineering Guangdong Laboratory (Guangzhou), Guangzhou, Guangdong, China; ^3^Department of Ocean Science, Hong Kong University of Science and Technology, Kowloon, Hong Kong SAR, China

**Keywords:** polyene pyrone, *streptomyces*, biosynthesis, polyketide, anti-neuroinflammatory

## Abstract

This study investigated the chemical composition and biosynthesis pathway of compounds produced by *Streptomyces sulphureus* DSM 40104. With the guild of molecular networking analysis, we isolated and identified six uncommon structural characteristics of compounds, including four newly discovered pyridinopyrones. Based on genomic analysis, we proposed a possible hybrid NRPS-PKS biosynthesis pathway for pyridinopyrones. Notably, this pathway starts with the use of nicotinic acid as the starting unit, which is a unique feature. Compounds **1**–**3** exhibited moderate anti-neuroinflammatory activity against LPS-induced BV-2 cell inflammation. Our study demonstrates the diversity of polyene pyrone compounds regarding their chemical structure and bioactivity while providing new insights into their biosynthesis pathway. These findings may lead to the development of new treatments for inflammation-related diseases.

## 1. Introduction

Pyridinopyrones were a class of natural products containing a pyrone ring and a pyridine ring linked by polyene chains. They were initially isolated from marine-derived *streptomyces* by the Fenical group (Fukuda et al., 2011). The structure of pyridinopyrones bears a substantial similarity to the aromatic polyene pyrones, which exhibit remarkable biological properties, including cytotoxicity (Clark et al., 2006; Hua et al., 2013b), anti-inflammatory effects (Hua et al., 2013a), mitochondrial oxidative phosphorylation inhibition (Lin et al., 2016) among others. The biosynthesis studies of aromatic polyene showed that it is produced by a modular type 1 PKS pathway (Peng et al., 2019). The broad range of bioactivities and the interesting biosynthesis studies associated with these compounds has sparked considerable interest in exploring new aromatic polyene pyrones.

Molecular networking has emerged as an efficient tool in natural product discovery to identify typically clustered compounds (Yang et al., 2013; Quinn et al., 2017). It involves using mass spectrometry to identify molecules in complex biological systems, especially in microbes (Watrous et al., 2012). In this process, compounds with similar structural features are grouped into a network based on the mass-to-charge ratio of their ionized forms (Schmid et al., 2021). Consequently, compound clusters are identified within the network, and these groups can then be further investigated to determine their chemical structures and biological activities. The approach has been used to discover bioactive compounds, such as antibiotics, from microbial sources (Nothias et al., 2018). Moreover, this technique has shown great promise in improving the efficiency of natural product discovery. Its application is expected to lead to the discovery of numerous bioactive compounds.

Through our ongoing effort to explore new compounds from *Streptomyces* (Tang et al., 2018; Liu et al., 2019, 2020; She et al., 2021), our research group has investigated the secondary metabolisms of the strain *Streptomyces sulphureus* DSM 40104 and uncovered four new pyridinopyrones with the guild of molecular networking analysis. Furthermore, we have investigated the anti-neuroinflammatory potential of the new pyridinopyrones.

## 2. Materials and methods

### 2.1. General experimental procedures

An ultra-high-performance liquid chromatography-mass spectrometry (UPLC-MS) analysis of metabolites was performed on an AB SCIEX LC-30A-Triple TOF 5600+ ESI MS system (AB SCIEX, Boston, MA, USA) equipped with a Shim-Pack Velox C18 column (2.7 μm, 150 mm × 2.1 mm, SHIMADZU, Japan), and a Thermo QE Focus instrument (Thermo Fisher Scientific, Waltham, MA, USA) with an Accucore AQ C18 column (2.6 μm, 150 mm × 2.1 mm, Thermo Fisher Scientific, Waltham, MA, USA). High-resolution electrospray ionization mass spectrometry (HRESIMS) data of novel metabolites were acquired by a Thermo Scientific Q Exactive mass spectrometer. Analytical high-performance liquid chromatography (HPLC) and semipreparative HPLC were carried out on an Agilent 1100 series system (Agilent Technologies Inc., Santa Clara, CA, USA) with a YMC-Pack ODS-A column (5 μm, 250 mm × 4.6 mm; 5 μm, 250 mm × 10 mm, YMC CO., LTD., Japan). Medium-pressure liquid chromatography (MPLC) was performed on a Buchi Medium pressure preparative chromatography (BUCHI Labor Technik AG, Switzerland) equipped with a medium-pressure column containing YMC reversed-phase (RP)-C_18_ silica gel. Gel chromatography was carried out on a gel column with Sephadex LH-20 (40–70 μm; Amersham Pharmacia Biotech AB, Sweden). ^1^H, ^13^C, and 2D NMR data were acquired on a Bruker Avance Neo 400 MHz apparatus (Bruker Corporation, Faellanden, Switzerland).

### 2.2. Bacterial material

The strain *S. sulphureus* DSM 40104 was purchased from Germany Leibniz-Institute DSMZ-German Collection of Microorganisms and Cell Cultures GmbH. The strain was stored in a 20% glycerol/water frozen tube at −80°C. The genome data were assembled by scaffold level and publication in GenBank with the accession number NZ_ARLC00000000.

### 2.3. Fermentation, extraction, and isolation

Strain DSM 40104 was activated and cultured by a Trypticase Soy Broth (TSB) medium in a thermostatic shaker (120 rpm) at 28°C for 1 week. We inoculated the DSM 40104 seed (20 ml) into a 1 L culture vessel containing 400 ml of SPY liquid medium with 100 × trace element and continued to culture in a rotatory shaker (120 rpm) at 28°C for 9 days. The SPY liquid medium included soluble starch (1%), peptone (0.2%), yeast extract (0.4%), and sodium chloride (0.5%). The trace elements were as follows: 49.8 mg FeCl_3_ in 100 ml + 1 ml of 0.1 M ZnCl_2_ +76.5 μl of 0.1 M CuCl_2_ + 21 μl of 0.2 M CoCl_2_ + 8.1 μl of 1 M MnCl_2_. After fermentation, 115 L of fermentation broth was filtered with gauze to obtain the mycelia and supernatant. The supernatant was extracted five times with an equal amount of ethyl acetate and evaporated under a vacuum to get the crude extract (20 g).

The crude extract (20 g) was segregated by MPLC equipped with an RP-18 column, and 13 components (fractions A–M) were obtained with a gradient elution of MeOH–H_2_O (30:70 → 100:0, v/v). Fractions E (342 mg), F (264 mg), and G (299 mg) were separated by using a Sephadex LH-20 column (MeOH) to get fractions E1–8, F1–6, and G1–7, respectively. Among these sub-fractions, six of them (fractions A, B, E5, F4, G5, G6) were submitted to the molecular networking analysis to guild for further purification. Through further purification by RP-C_18_ HPLC with a semipreparative column (40%, MeCN–H_2_O, 2 ml/min), **1** (*t*_R_ = 51.0 min, 2.4 mg) was isolated from fraction E5. Then, fraction F4 was subjected to RP-C_18_ HPLC (45%, MeCN–H_2_O, 2 ml/min) to obtain **2** (*t*_R_ = 52.4 min, 3.3 mg) and **6** (*t*_R_ = 58.6 min, 7 mg). Fraction G5 was further purified by RP-C_18_ HPLC with an analytical column (45%, MeCN–H_2_O, 1 ml/min) to obtain **3** (*t*_R_ = 34.9 min, 3.8 mg), and fraction G6 was further purified by RP-C18 HPLC (50%, MeCN–H_2_O, 2 ml/min) to obtain **4** (*t*_R_ = 37 min, 11.7 mg) and **5** (*t*_R_ = 45.7 min, 13.9 mg).

*Pyridinopyrone E (**1**)*: amorphous yellow solid; UV (MeOH) λ_max_ 380 nm, 290 nm, 240 nm; ^1^H and ^13^C NMR, see Table 1; HRESIMS [M + H]^+^ *m/z* 296.1273 (calculated for C_18_H_17_NO_3_, 296.1287).

**Table 1:** NMR spectroscopic data for the pyridinopyrone E (**1**), F (**2**) (400 MHz for ^1^H, 100 MHz for ^13^C in DMSO-*d*_6_).

**No**.	**1**		**2**	
	δ_C_	δ_H_ **mult (*****J*** **in Hz)**	δ_C_	δ_H_ **mult (*****J*** **in Hz)**
1	162.6, C		163.4, C	
2	88.6, CH	5.65, s	100.8, C	
3	171.0, C		165.9, C	
4	99.0, CH	6.33, s	97.0, CH	6.68, s
5	159.9, C		156.8, C	
6	126.9, C		123.0, CH	6.39, d (15.2)
7	130.9, CH	7.07, d (11.4)	134.2, CH	7.08, dd (15.2, 11.2)
8	129.8, CH	6.9, m	132.4, CH	6.57, m
9	138.2, CH	6.87, m	138.1, CH	6.78, m
10	131.2, CH	7.27, dd (15.8, 9.7)	134.3, CH	6.61, m
11	130.9, CH	6.79, d (15.8)	135.7, CH	6.63, m
12	N/A	N/A	131.1, CH	7.17, dd (15.8, 9.9)
13	N/A	N/A	130.1, CH	6.74, d (15.8)
2′	148.3, CH	8.69, s	148.3, CH	8.67, s
3′	132.6, C		133.2, C	
4′	132.7, CH	7.95, d (8.0)	132.7, CH	7.94, dt (8.0, 2.0)
5′	123.9, CH	7.39, dd (8.0, 4.8)	123.8, CH	7.37, dd (8.0, 4.7)
6′	148.7, CH	8.44, d (4.8)	148.5, CH	8.42, d (4.7)
3-OCH_3_	56.5, CH_3_	3.84, s	56.8, CH_3_	3.9, s
2-CH_3_	N/A	N/A	8.9, CH_3_	1.81, s
6-CH_3_	12.4, CH_3_	2.01, s	N/A	N/A

N/A, not available.

*Pyridinopyrone F (**2**)*: amorphous yellow solid; UV (MeOH) λ_max_ 430 nm, 400 nm, 320 nm, 255 nm; ^1^H and ^13^C NMR, see Table 1; HRESIMS [M+H]^+^ *m/z* 322.1440 (calculated for C_20_H_19_NO_3_, 322.1443).

*Pyridinopyrone G (**3**)*: amorphous yellow solid; UV (MeOH) λ_max_ 430 nm, 400 nm, 320 nm, 260 nm; ^1^H and ^13^C NMR, see Table 2; HRESIMS [M+H]^+^ *m/z* 336.1595 (calculated for C_21_H_21_NO_3_, 336.1600).

**Table 2:** NMR spectroscopic data for the pyridinopyrone G (**3**), H (**4**) (400 MHz for ^1^H, 100 MHz for ^13^C in DMSO-*d*_6_).

**No**.	**3**		**4**	
	δ_C_	δ_H_ **mult (*****J*** **in Hz)**	δ_C_	δ_H_ **mult (*****J*** **in Hz)**
1	163.4, C		162.6, C	
2	100.6, C		88.5, CH	5.62, s
3	166.0, C		170.9, C	
4	94.1, CH	6.6, s	98.7, CH	6.30, s
5	158.7, C		160.0, C	
6	126.9, C		126.5, C	
7	130.8, C	7.07, d (11.6)	131.2, CH	7.04, d (11.4)
8	129.4, CH	6.84, m	129.3, CH	6.81, m
9	138.1, CH	6.78, m	138.4, CH	6.79, m
10	134.6, CH	6.64, m	134.5, CH	6.65, m
11	135.3, CH	6.64, m	135.5, CH	6.65, m
12	131.1, CH	7.18, m	131.0, CH	7.17, m
13	129.9, CH	6.73, d (15.6)	130.0, CH	6.74, d (15.6)
2′	148.3, CH	8.67, s	148.3, CH	8.67, s
3′	133.6, C		133.5, C	
4′	132.6, CH	7.95, dt (8.0, 2.0)	132.7, CH	7.95, dt (7.9, 2.0)
5′	123.8, CH	7.36, dd (8.0, 4.6)	123.8, CH	7.36, dd (7.9, 4.7)
6′	148.4, CH	8.42, d (4.6)	148.5, CH	8.42, d (4.7)
3-OCH_3_	56.8, CH_3_	3.96, s	56.4, CH_3_	3.81, s
2-CH_3_	8.8, CH_3_	1.81, s	N/A	N/A
6-CH_3_	12.5, CH_3_	2.07, s	12.4, CH_3_	2.00, s

N/A, not available.

*Pyridinopyrone H (**4**)*: amorphous yellow solid; UV (MeOH) λ_max_ 430 nm, 400 nm, 320 nm, 255 nm; ^1^H and ^13^C NMR, see Table 2; HRESIMS [M+H]^+^ *m/z* 322.1440 (calculated for C_20_H_19_NO_3_, 322.1443).

### 2.4. Molecular networking construction

After separation by MPLC and Sephadex LH-20 column, six parts with enough amount for further purification were selected for further molecular networking construction based on HPLC analysis and UPLC-MS data results. The six parts were fractions A, B, E5, F4, G5, and G6.

The samples were handled by dissolving them in methanol to a concentration of 1 mg/ml. Two methods were used for UPLC-MS analysis. The fraction A, B, and E were analysis base on the following gradient: 0–2 min, 2% B; 3–8 min, 2%−50% B; 8–18 min, 50%−100% B; 18–20 min, 100% B; 20–22 min, 100%-2% B; 22–27 min, 2% B, in which mobile phase A was water and mobile phase B was acetonitrile. The gradient elution approach of fractions F4, G5, and G6 was the water (A)–methanol (B) system, and the details were as follows: 0–5 min, 5% B; 5–15 min, 100% B; 15–18 min, 100% B; 18–19 min, 5% B. The flow rate was set at 0.3 ml/min with an injection volume of 1 μl. The MS detector was set to a positive mode.

By using the UPLC-MS data of fractions A, B, E5, F4, G5, and G6, the molecular networking of the six fractions was constructed on the Global Natural Products Social Molecular Networking (GNPS) platform (gnps.ucsd.edu) based on a previously reported method (Liu et al., 2020). Specifically, the raw data files were converted from .wiff standard data format into .mzML format files by MSConvert software to support uploading the files to GNPS. Then, the converted files were submitted to the GNPS platform by 8UFTP software. The molecular networking was constructed using the online workflow at GNPS with a fragment ion mass tolerance of 0.5 Da. Cytoscape 3.9.0 was used to visualize and analyze the molecular network.

### 2.5. Antibacterial bioassay

The six compounds were estimated *in vitro* for their antibacterial activity against *Escherichia coli* (Gram-negative bacteria) and methicillin-resistant *Staphylococcus aureus* (MRSA, Gram-positive bacteria). The minimum inhibitory concentration (MIC) was used to evaluate the antibacterial activity of these compounds.

The double dilution method determined the inhibitory activity of compounds against the tested bacteria according to a previously reported method (Su et al., 2017; Lin et al., 2021a). Briefly, the six samples were configured with DMSO into a solution with a concentration of 25 mM. There were two kinds of antibiotics, vancomycin, and kanamycin, used as a positive control for MRSA and *E. coli*, respectively. Approximately 0.5% of DMSO worked as a negative control. MRSA and *E. coli* stored in a 20% glycerol/water frozen tube at −80°C were activated with MHB medium in a shaker at 180 rpm/min and 37°C until the strain entered the logarithmic growth phase. The bacteria in the logarithmic growth stage were diluted with MHB medium into 2 × 10^6^ CFU/ml and added into a 96-well plate, 100 μl/well. Approximately 100 μl of sample solution with different concentrations (100 μM, 50.0 μM, 25.0 μM, 12.5 μM, 6.25 μM, and 3.12 μM) diluted by MHB medium was added into a 96-well plate, and each sample with diverse concentration and each control consisted of three replicates. Then, the 96-well plates were placed in a 37°C incubator for 16 h. If the bacteria grew up, the bacterial solution would become turbid, and the compound concentration inhibiting the growth of bacteria was the MIC of the compound. Meanwhile, the bacterial solution was determined by the absorbance at 600 nm with a microplate recorder (BioTek Epoch, BioTek Instruments. Inc., Winooski, VT, USA) to verify the correctness of the MIC.

### 2.6. Anti-neuroinflammatory activities

According to the previously reported literature, the anti-neuroinflammatory activities of compounds were evaluated by nitric oxide (NO) production inhibitory assays using BV-2 murine microglial cells (Tang et al., 2019; Lin et al., 2021b; Xu et al., 2022). The BV-2 cells should be resuscitated, cultured in DMEM medium with 10% FBS and 1% penicillin–streptomycin solution, and placed in an incubator with 5% CO_2_ at 37°C. In the logarithmic growth period, 100 μl of BV-2 cells were seeded into a 96-well plate with a 2 × 10^5^ cells/ml concentration. After 24 hours incubation at 37°C, removing the supernatant, the prepared compounds with different concentrations (50 μM, 25 μM, 12.5 μM, 6.25 μM, 3.125 μM) and LPS (1 μg/ml) were added into 96-well plate, respectively and continued to culture for 24 h at 37°C. LPS and dexamethasone were regarded as positive controls, and LPS and DMSO were worked as negative controls. Each concentration and control were treated with three sets of repetitions.

The production of NO in BV-2 cells culture medium was measured with a NO assay kit, Griess kit. Specifically, 50 μl of BV-2 cells culture medium supernatant was transferred to a new 96-well plate. Then, 50 μl of Griess reagent I and II were added sequentially to the above supernatant. Then, the absorbance of the mixture at 540 nm was measured with a microplate reader. The standard curve of a series of sodium nitrite standard solutions calculated the nitrite concentration in the cell culture solution. IC_50_ values of each compound referred to the concentration of the compound, which reduced 50% of NO production and was calculated according to the standard curve.

## 3. Results and discussion

**Figure 1:**
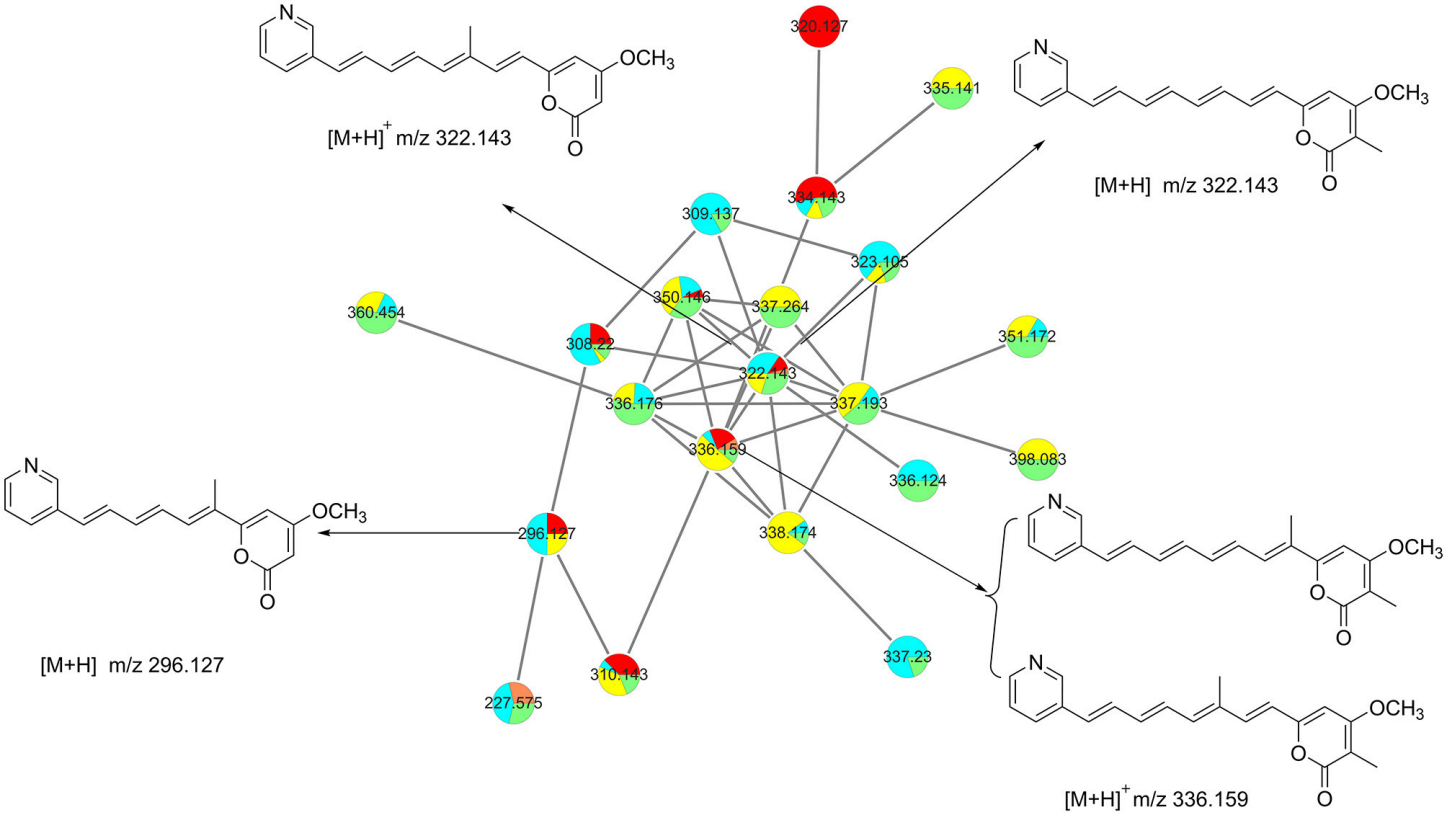
Molecular networking analysis guiding the isolation of pyridinopyrones.

Based on the six fractions (A, B, E5, F4, G5, and G6), we have constructed a molecular network with hundreds of grouped compounds (Supplementary Figure S1). Among these, one of the groups with a molecular weight of approximately 322 attracted our attention. Further analysis of the molecular weight of these compounds, we have identified a rare kind of compound named pyridinopyrones (Figure 1). Until now, only four of these compounds have been reported (Fukuda et al., 2011; Hou et al., 2012). The chemical structure of this compound is characterized by a combination of a pyrone ring and a pyridine ring, with a polyene chain connecting the two rings. Compared with HRMS data from the previously published references, the molecular network exhibits more pyridinopyrones analogously connected to the same cluster. With the guide of molecular networking, we investigated the fractions that may possess new pyridinopyrones and identified six compounds.

Pyridinopyrone E (**1**) was isolated by semi-preparation HPLC as an amorphous yellow solid. The molecular formula was determined to be C_18_H_17_NO_3_ ([M+H]^+^ *m/z* 296.1273) based on high-resolution ESIMS, indicating 11 degrees of unsaturation. The UV absorption of 380 nm indicated that the high degree of unsaturation might be due to the extended conjugated group. The ^1^H NMR spectrum of **1** showed one methyl (δ_H_ 2.01, s), one methoxy (δ_H_ 3.84, s), and 11 olefinic/aromatic methine signals. The ^13^C NMR spectrum displayed 18 resolved signals, which were classified as one methyl (δ_C_ 12.4), one methoxy (δ_C_ 56.5), 11 sp^2^ methine, and five quaternary carbons, including two oxygenated carbons and one ester carbonyl (Table 1).

**Figure 2:**
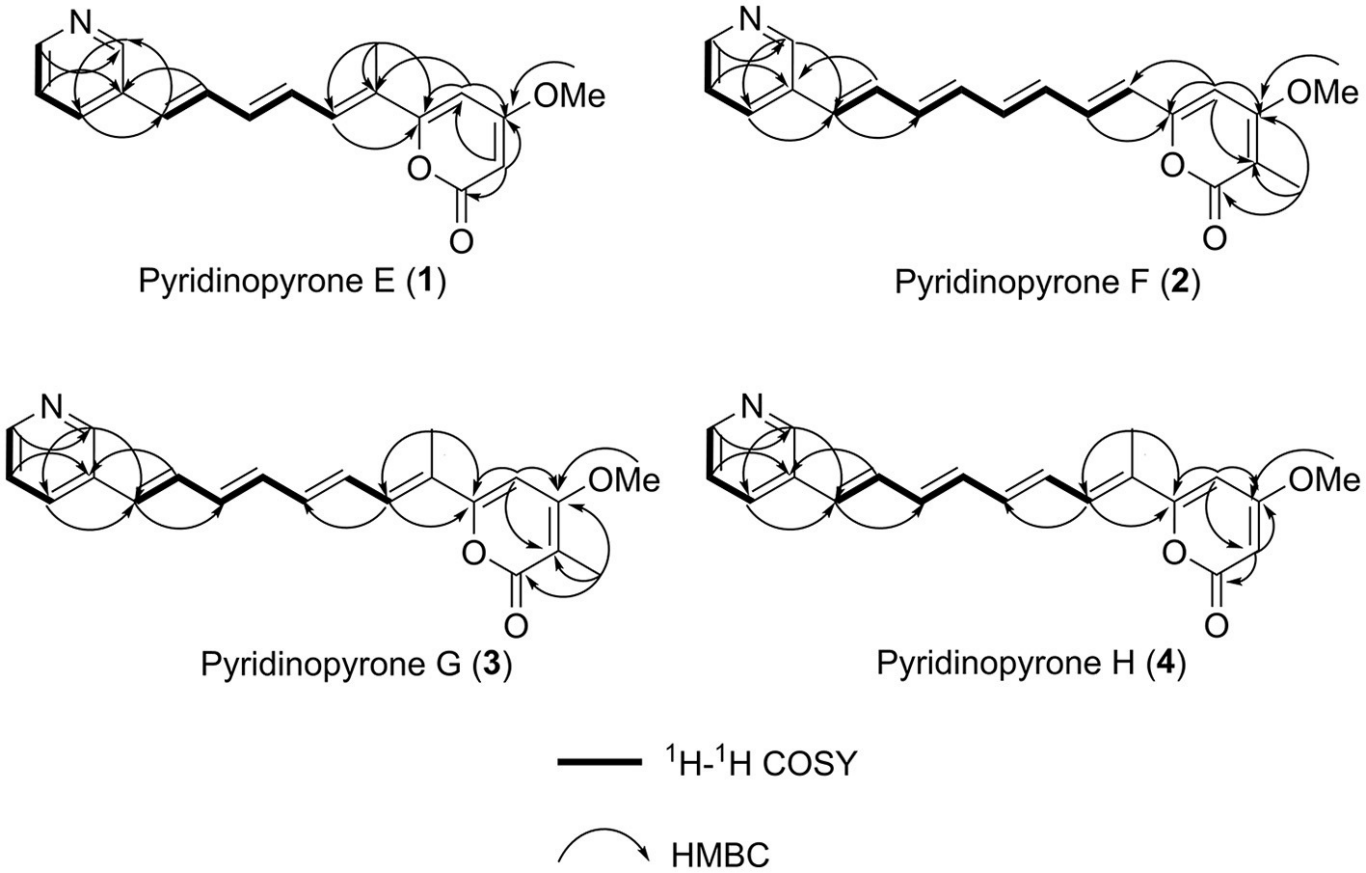
^1^H–^1^H COSY correlations and key HMBC correlations used to establish the structures of compounds (**1**–**4**).

**Figure 3:**
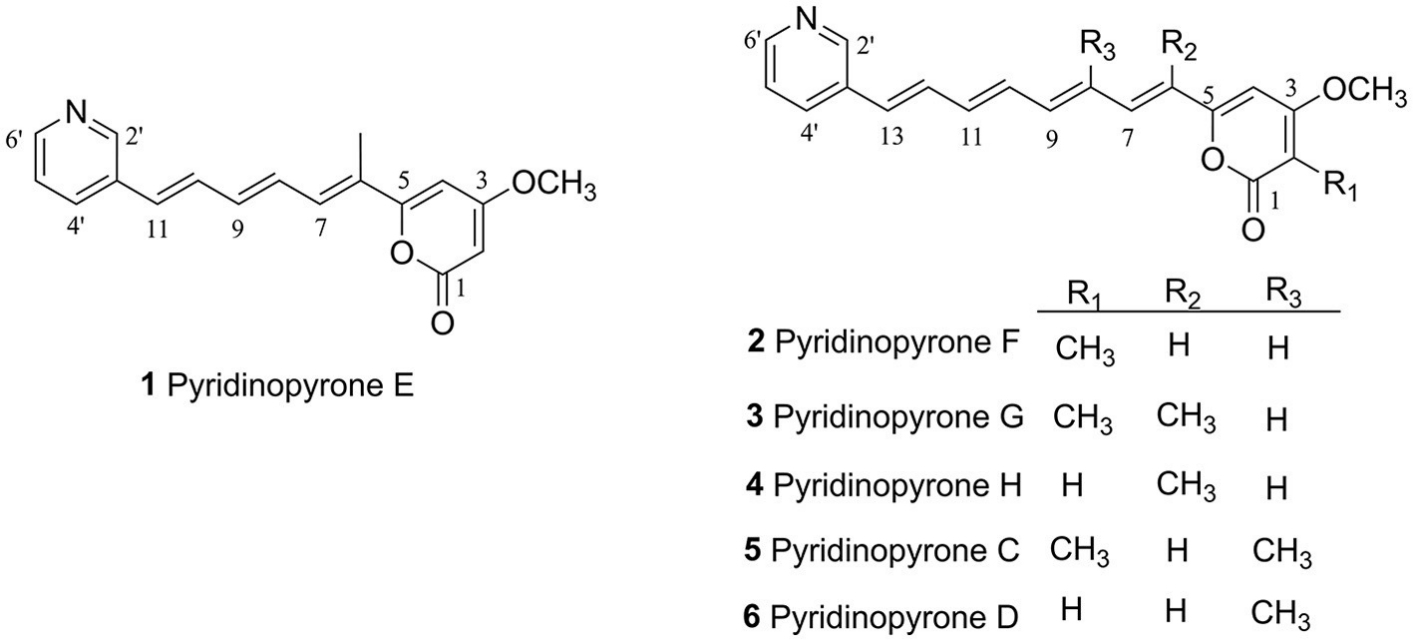
Chemical structure of compounds (**1**–**6**).

The ^1^H–^1^H COSY NMR data assigned two partial structures, H-7/H-8/H-9/H-10/H-11 and H-4′/H-5′/H-6′. Considering the chemical shifts and vicinal coupling information, the moiety of H-7/H-8/H-9/H-10/H-11 was established as a triene unit. Analysis of HMBC spectrum data gave further connected information about these two moieties. The correlation of H-6′ (δ_H_ 8.44) to C-2′ (δ_C_ 148.3), H-5′ (δ_H_ 7.39) to C-3′ (δ_C_ 132.6), and H-2′ (δ_H_ 8.69) to C-4′ (δ_C_ 132.7) indicated the existence of a pyridine ring. The cross-peaks from H-4′ (δ_H_ 7.95) to C-11 (δ_C_ 130.9), H-11 (δ_H_ 6.79) to C-2′, and H-10 (δ_H_ 7.27) to C-3′revealed the connection of triene moiety to the pyridine ring at position 3′. The HMBC correlations from H-2 (δ_H_ 5.65) to C-1 (δ_C_ 162.6), C-3 (δ_C_ 171.0), and C-4 (δ_C_ 99.0), from H-4 (δ_H_ 6.33) to C-3, and C-5 (δ_C_ 159.9) suggested the connective of C-1 to C-5 (Figure 2). The downfield chemical shift of C-5 indicated the connection of oxygen atoms. Thus, the existence of a pyrone unit in **1** was confirmed. The HMBC correlation from the 3-OCH_3_ protons to C-3 supported the connective of a methoxy group to pyrone at C-3. Further HMBC correlations from H-7 (δ_H_ 7.07) to C-5, H-4 (δ_H_ 6.33) to C-6, and 6-CH_3_ (δ_H_ 2.01) to C-5, C-6, and C-7 indicated the triene unit was linked at the C-5 position of the pyrone ring and the methyl group was connected at C-6. The triene unit was assigned all *E* geometrical configurations from the coupling constants (*J*_10_, _11_ = 15.8) and by comparison with similar compounds. Overall, the chemical structure of compound **1** was determined, as shown in Figure 3.

Pyridinopyrone F (**2**) was obtained as an amorphous yellow solid. Based on HRESIMS data, the molecular weight of **2** was 322.1440, and molecular formula was characterized as C_20_H_19_NO_3_, which was exactly the same as the pyridinopyrone A. By comparison of all 1D NMR data of compound **2** with that from pyridinopyrone A, the difference between these two compounds was the methyl group location. The 2D NMR spectra data of **2** showed the existence of pyridine and an α-pyrone ring. The cross-peak of H-6/H-7/H-8/H-9/H-10/H-11/H-12/H-13 observed in the ^1^H–^1^H COSY spectrum indicated the existence of a tetraene group (Figure 2). The HMBC correlations of H-2′ (δ_H_ 8.67) to C-4′ (δ_C_ 132.7) and C-13 (δ_C_ 130.1), H-4′ (δ_H_ 7.94) to C-13, and H-12 (δ_H_ 7.17) to C-3′ (δ_C_ 133.2) demonstrated the connection of tetraene moiety via C-3′ of the pyridine ring. The HMBC correlations of H-7 (δ_H_ 7.08) to C-5 (δ_C_ 156.8), H-4 (δ_H_ 6.68) to C-5, and C-6 (δ_C_ 123.0) allowed the assembly of the tetraene group and the α-pyrone rings through C-5. The attachment of methyl groups at C-2 was confirmed by the HMBC correlations of 2-CH_3_ (δ_H_ 1.81) protons to C-1 (δ_C_ 163.4), C-2 (δ_C_ 100.8), and C-3 (δ_C_ 165.9). The 3-OCH_3_ (δ_H_ 3.90) protons to C-3 (δ_C_ 56.8) indicated the linkage of the methoxy group at the C-3 position. The tetraene unit was assigned all *E* geometrical configurations base on the coupling constants (*J*_6,7_ = 15.2, *J*_12,13_ = 15.8) and the comparison with similar compounds.

Pyridinopyrone G (**3**) was obtained as an amorphous yellow solid with the HRESIMS [M+H]^+^ *m/z* 336.1595 (calculated for C_21_H_21_NO_3_, 336.1600). The molecular weight of **3** was exactly the same as pyridinopyrone C. By comparison of the 1D and 2D NMR data, we found that the difference between **3** and pyridinopyrone C was the position of one methyl group. The HMBC correlations of 6-CH_3_ (δ_H_ 2.07) protons to C-5 (δ_C_ 158.7) and C-7 (δ_C_ 130.8) confirmed the attachment of methyl at the C-6 position (Figure 2). Thus, the structure of **3** was determined, as shown in Figure 3.

Pyridinopyrone H (**4**) was isolated as an amorphous yellow solid. The molecular formula was determined as C_20_H_19_NO_3_ based on the HRESIMS of [M+H]^+^ *m/z* 322.1440. The 1D and 2D NMR spectra data showed that the structure of **3** was the same as compound **2** except for the methyl group's position. The olefinic methine signal at δ 5.62 and the HMBC correlations of H-2 (δ_H_ 5.62) to C-1 (δ_C_ 162.6) and C-3 (δ_C_ 170.9) indicated the absence of the methyl group at C-2 (Figure 2). The HMBC correlation of 6-CH_3_ (δ_H_ 2.00) to the C-5 (δ_C_ 160.0) and C-6 (δ_C_ 126.5) confirmed the position of the methyl group at C-6. Overall, the structure of **4** was determined, as shown in Figure 3.

Compounds **5** and **6** were determined to be pyridinopyrones C and D (Fukuda et al., 2011; Hou et al., 2012), respectively, based on the analysis of NMR spectra and comparison of literature.

**Figure 4:**
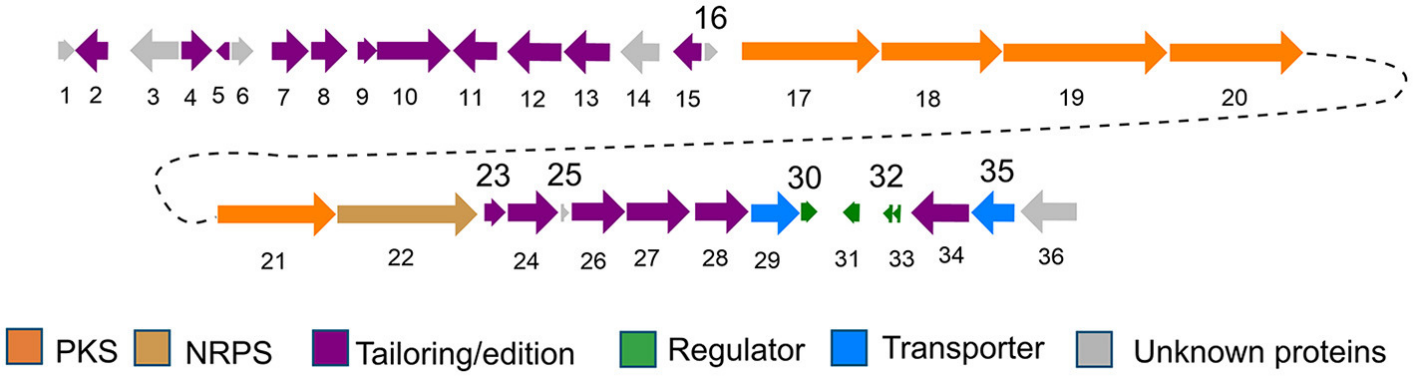
Gene organization of the pyridinopyrones (*pyi*) BGC (**1**–**6**).

The structure of pyridinopyrones showed substantial similarity to polyene pyrones which are rare in bacteria rather than fungi (Clark et al., 2006; Liu et al., 2011; Lin et al., 2016). In the case of these polyenylpyrroles, the end of the group is pyrrole or furan rather than pyridine. The biosynthesis pathway of the pyridinopyrones was studied by the Fenical group through initial labeling studies. The result showed that the pyridinopyrones were derived from acetate-extended nicotinic acid and produced by an iterative PKS (Fukuda et al., 2011; Woerly et al., 2014). We have examined the draft genome data of strain DSM 40104 and gotten 27 gene clusters (Supplementary Table S1). Among these gene clusters, a hybrid modular type 1 polyketide synthases (PKSs) and non-ribosomal peptide synthetase (NRPs) gene cluster attracted our attention. There are 36 open reading frames (ORFs) in this gene cluster, including five PKS genes, a non-ribosomal peptides (NRPS) gene, four regulators, two transporters, and 24 dispersed genes (Figure 4 and Supplementary Table S2).

**Figure 5:**
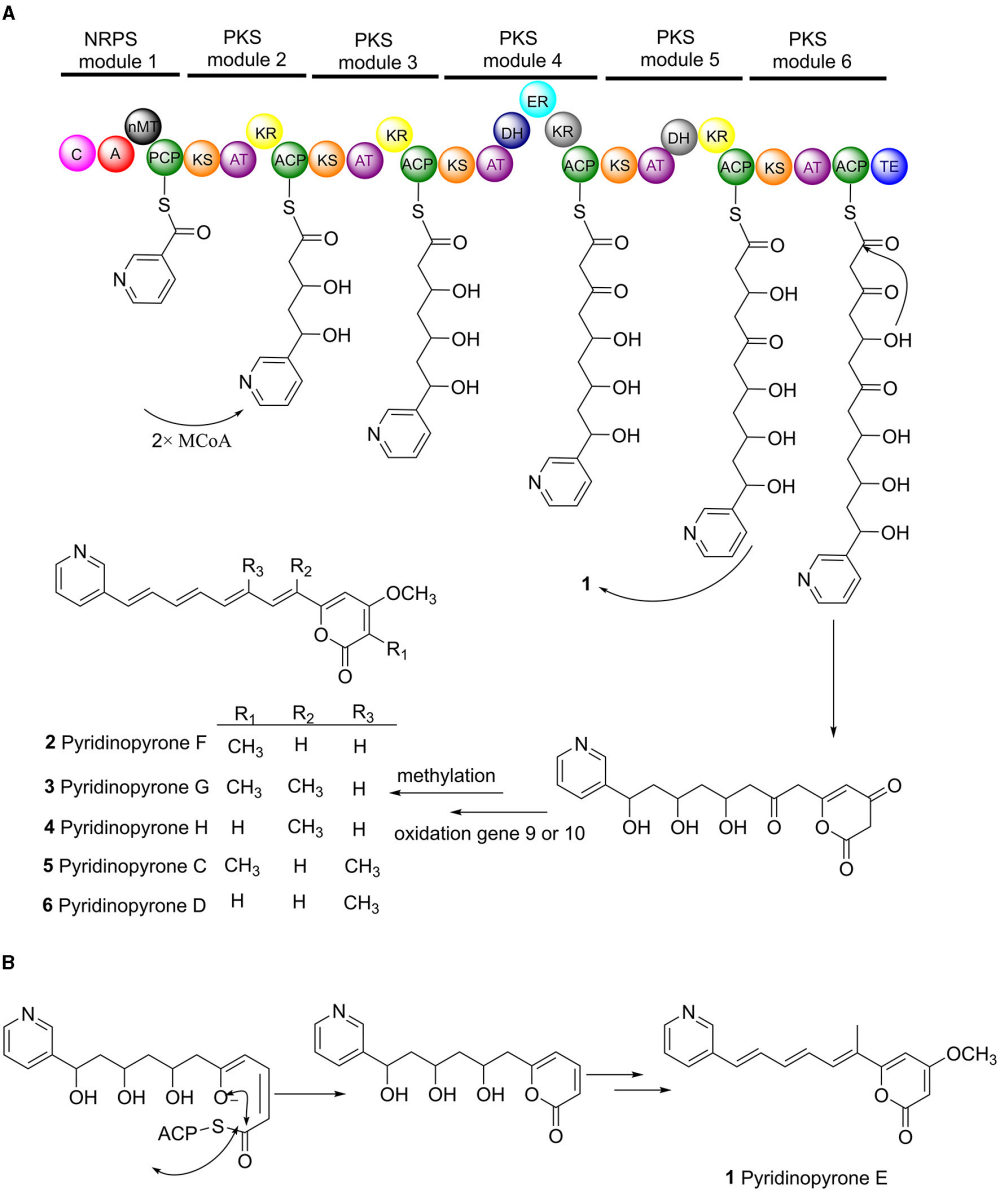
Proposed biosynthesis pathway of pyridinopyrones (**1**–**6**). Pyi PKS organization and deduced assembly of **1**–**6 (A)**. The formation of pyrone ring in compound **1 (B)**.

The biosynthesis pathway of pyridinopyrones was proposed based on the bioinformatic analysis and the literature comparison. The hybrid gene cluster *pyi* was proposed to be responsible for the production of pyridinopyrones. The adenylation (A-) domain was unique, and there were no specific amino acid residues can be recognized by this A-domain base on the blasted analysis (Supplementary Figure S5). We proposed that the nicotinic acid might be activated by the A-domain as a starter unit and loaded onto the peptidyl carrier (PCP-) domain. The activated nicotinic acid was propagated by five PKS modules with acyltransferase (AT) domain selecting and loading the malonyl extender unit. The polyketide chain is passed from one module to another until it is released by the thioesterase (TE) domain (Figure 5A). The isolated pyridinopyrones possess different chain lengths indicating the different number of modules adopted during the process. Compound **1** was released from modular five, and compounds **2**–**6** were released from modular six by forming the pyrone ring (Figure 5B). The following oxidation and methylation lead to the final production of **1**–**6**.

The proposed biosynthesis pathway showed low similarity with the known gene clusters. The A-domain is responsible for recruiting the amino acid monomers, which could be used to predict the putative substrates (Niquille et al., 2018). The nicotinic acid as a start unit in the NRPS module is interesting. The loss of single-domain function in modular 4 and 5 alter the final chemical structure of the products (Peng et al., 2019). The function of ketoreductase (KR) in module 4 and the dehydrogenase (DH) in module 5 were inactive (Supplementary Figures S3, S4), influencing the degree of β-keto processing.

The pyridinopyrones bear a substantial similarity to the polyenylpyrroles and the polyenylfurans, which have been reported to possess anti-HIV activity (Zhong et al., 2022), cytotoxic activity (Clark et al., 2006), and cytoprotective activity (Yamagishi et al., 1993). Given these literature reports, we have tested the antibacterial activity, taking vancomycin as a positive control. However, all test compounds showed no antibacterial activity in gram-positive and gram-negative bacteria.

**Figure 6:**
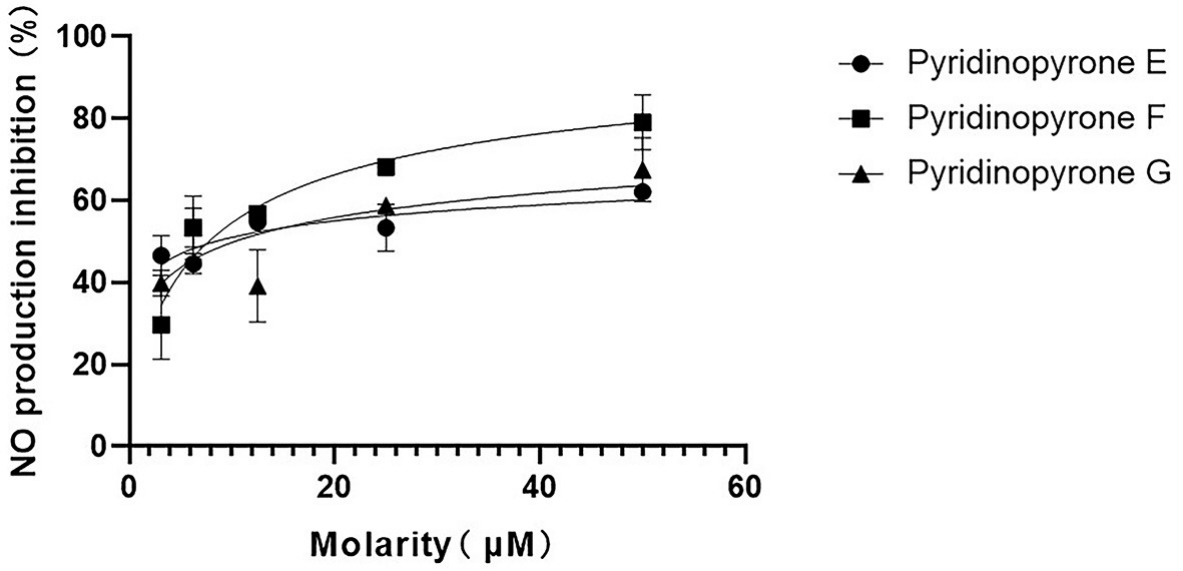
Inhibitory activity of compounds **1**–**3** with different concentrations on NO production in LPS-induced BV-2 cells.

In addition, we have conducted the anti-neuroinflammatory activity of compounds **1**–**3**. The result showed that compounds **1**–**3** exhibited significant anti-neuroinflammatory activity against the lipopolysaccharide (LPS) induced BV-2 cell inflammation at a concentration of 50 μM (Supplementary Figure S6). We further detected the inhibition of the NO production of all three new pyridinopyrones at different concentrations. The IC_50_ value of compounds **1**–**3** was 8.384 μM, 7.739 μM, and 10.28 μM, respectively (Figure 6). The dexamethasone (DXMS) was considered a positive control with an IC_50_ value of 6.854 μM (Supplementary Figure S7).

In conclusion, our study examined the chemical composition of compounds produced by *S. sulphureus* DSM 40104. With the assistance of molecular networking analysis, we were able to isolate and identify six unique structural characteristics of compounds. Among these were four newly discovered pyridinopyrones. We also conducted a genomic analysis to propose the possible biosynthesis pathway for pyridinopyrones. This hybrid NRPS-PKS pathway was found to have an unusual A-domain that can recognize nicotinic acid. Furthermore, we discovered that compounds **1**–**3** exhibited significant anti-neuroinflammatory activity against the LPS-induced BV-2 cell inflammation. Overall, our findings demonstrate the diversity of polyene pyrone compounds regarding the chemical structure and bioactivity while providing new insights into their biosynthesis pathway.

## Data availability statement

The datasets presented in this study can be found in online repositories. The names of the repository/repositories and accession number(s) can be found in the article/Supplementary material.

## Author contributions

P-YQ and L-LL contributed to the conception and design of the study. JH and P-ML performed the experiments and collected the experimental data. Z-XW performed part of the data analysis. L-LL wrote the first draft of the manuscript. JH wrote sections of the manuscript. All authors contributed to the manuscript revision and read and approved the submitted version.
