# Supplementary material for: Structural identification of pyridinopyrone compounds with anti-neuroinflammatory activity from streptomyces sulphureus DSM 40104
Source: Front Microbiol. 2023 Jun 1;14:1205118. doi: 10.3389/fmicb.2023.1205118 (PMC10268602; doi:10.3389/fmicb.2023.1205118)

Supplementary Material

Structural Identification of Pyridinopyrone Compounds with Anti-Neuroinflammatory Activity from *Streptomyces Sulphureus* DSM 40104

Juan Hu[1], Zi-Xuan Wang[1], Pei-Meng Li[1], Pei-Yuan Qian[2, 3*], Ling-Li Liu[1, 2*]

[1] Shaanxi Key Laboratory of Natural Products & Chemical Biology, College of Chemistry & Pharmacy, Northwest A&F University, Yangling 712100, Shaanxi, People's Republic of China
[2] Southern Marine Science and Engineering Guangdong Laboratory (Guangzhou), Nansha, Guangzhou, China
[3] Department of Ocean Science, Hong Kong University of Science and Technology, Hong Kong, China

*** Correspondence:**

Pei-Yuan Qian, Email: boqianpy@ust.hk

Ling-Li Liu, Email: leonie@nwsuaf.edu.cn

Index

Table S1 The gene clusters of strain *Streptomyces Sulphureus* DSM 40104 proposed by antiSMASH 3
Table S2. Annotation and homologues of genes in *pyi* 5
Figure S1. The molecular network obtained by combining the LC-MS/MS analyses of six fractions of extracts from strain DSM 40104. 7
Figure S2. Target cluster extract from the molecular network 8
Figure S3. The alignment result of all KR domains in the *pyi* gene cluster 9
Figure S4. The alignment result of both DH domains in the *pyi* gene cluster 9
Figure S5. The conserved domains analysis of A domains in the *pyi* gene cluster 9
Figure S6. Inhibitory activity of compounds 1~3 (50 μM) on NO production in LPS-induced BV-2 cells 10
Figure S7. Inhibitory activity of dexamethasone with different concentrations on NO production in LPS-induced BV-2 cells 11
Figure S8. ^{1}H NMR spectrum of pyridinopyrone E (**1**) (400 MHz, DMSO-d_6).. 12
Figure S9. ^{13}C NMR spectrum of pyridinopyrone E (**1**) (400 MHz, DMSO-d_6). 13
Figure S10. HSQC spectrum of pyridinopyrone E (**1**) (DMSO-d_6) 14
Figure S11. ^{1}H-^{1}H COSY spectrum of pyridinopyrone E (**1**) (DMSO-d_6) 15
Figure S12. HMBC spectrum of pyridinopyrone E (**1**) (DMSO-d_6) 16
Figure S13. HRESIMS spectrum of pyridinopyrone E (**1**) 17
Figure S14. UV spectrum of pyridinopyrone E (**1**) 18
Figure S15. ^{1}H NMR spectrum of pyridinopyrone F (**2**) (400 MHz, DMSO-d_6) 19
Figure S16. ^{13}C NMR spectrum of pyridinopyrone F (**2**) (400 MHz, DMSO-d_6) 20
Figure S17. HSQC spectrum of pyridinopyrone F (**2**) (DMSO-d_6) 21
Figure S18. ^{1}H-^{1}H COSY spectrum of pyridinopyrone F (**2**) (DMSO-d_6) 22
Figure S19. HMBC spectrum of pyridinopyrone F (**2**) (DMSO-d_6) 23
Figure S20. HRESIMS spectrum of pyridinopyrone F (**2**) 24
Figure S21. UV spectrum of pyridinopyrone F (**2**) 25
Figure S22. ^{1}H NMR spectrum of pyridinopyrone G (**3**) (400 MHz, DMSO-d_6)26
Figure S23. ^{13}C NMR spectrum of pyridinopyrone G (**3**) (400 MHz, DMSO-d_6) 27
Figure S24. ^{1}H-^{1}H COSY spectrum of pyridinopyrone G (**3**) (DMSO-d_6) 28
Figure S25. HSQC spectrum of pyridinopyrone G (**3**) (DMSO-d_6) 29
Figure S26. HMBC spectrum of pyridinopyrone G (**3**) (DMSO-d_6) 30
Figure S27. HRESIMS spectrum of pyridinopyrone G (**3**) 31
Figure S28. UV spectrum of pyridinopyrone G (**3**) 32
Figure S29. ^{1}H NMR spectrum of pyridinopyrone H (**4**) (400 MHz, DMSO-d_6)33
Figure S30. ^{13}C NMR spectrum of pyridinopyrone H (**4**) (400 MHz, DMSO-d_6) 34
Figure S31. ^{1}H-^{1}H COSY spectrum of pyridinopyrone H (**4**) (DMSO-d_6) 35
Figure S32. HSQC spectrum of pyridinopyrone H (**4**) (DMSO-d_6) 36
Figure S33. HMBC spectrum of pyridinopyrone H (**4**) (DMSO-d_6) 37
Figure S34. HRESIMS spectrum of pyridinopyrone H (**4**) 38
Figure S35. UV spectrum of pyridinopyrone H (**4**) 39

Table S1 The gene clusters of strain *Streptomyces Sulphureus* DSM 40104 proposed by antiSMASH

No.	Region	Type	From	To	Most similar known cluster	Similarity
1	1.1	siderophore	1	9535	desferrioxamine E	100%
2	1.2	T2PKS	170089	240643	WS79089A,hexaricin B, hexaricin C	18%
3	1.3	T3PKS	569454	610569	alkylresorcinol	100%
4	1.4	T2PKS	875755	939081	julichrome Q3-3, Q3-5	37%
5	2.1	Lanthipeptide-class-iii	61388	93709	chejuenolide A/B	7%
6	2.2	linaridin	122267	142818	siomycin A	7%
7	2.3	lassopeptide	267516	289027	anantin C	75%
8	2.4	NRPS,T1PKS	455124	529144	phthoxazolin	9%
9	3.1	ectoine	53961	64365	ectoine	100%
10	4.1	T1PKS	89788	168918	X-14547	30%
11	4.2	Lanthipeptide-class-i	527160	574350	paromomycin	7%
12	4.3	terpene	584547	611217	hopene	61%
13	5.1	T1PKS/NRPS	186651	287339	kirromycin	15%
14	6.1	siderophore	53692	68255	ficellomycin	3%
15	6.2	T1PKS	330686	378047	actinoallolide A	20%
16	7.1	Lanthipeptide-class-i	10722	35292	planosporicin	86%
17	7.2	LAP,thiopeptide	51102	83722	diazepinomicin	7%
18	7.3	NRPS	207984	258573	WS9326	5%
19	9.1	PKS-like,T3PKS	101452	148827	viguiepinol	26%
20	9.2	T1PKS	306804	349537	armeniaspirols	24%
21	10.1	NRPS	223882	266554	coelibactin	36%
22	11.1	T1PKS/NRPS	101067	173007	microtermolide A	13%
23	15.1	nucleoside	41577	61951	thiolutin	12%

No.	Region	Type	Size (NT)		Most similar known cluster	Similarity
24	16.1	T2PKS	1	56609	resistomycin/resistoflavine	88%
25	18.1	RRE-containing	62565	72882	bottromycin A2	12%
26	19.1	NRPS	3711	47640	bacillibactin	38%
27	24.1	RiPP-like	1	9160	bottromycin D	72%

Table S2. Annotation and homologues of genes in *pyi*

genes	Size (AA)	Product	Source	Identity/ Positives	Accession Number
1	145	hypothetical protein	*Streptomyces xiaopingdaonensis*	90/91	WP_033196418
2	293	virginiamycin B lyase	*Streptomyces lushanensis*	72/82	WP_079125586
3	415	hypothetical protein	*Streptomyces xiaopingdaonensis*	96/97	WP_016910516
4	286	endo alpha-1,4 polygalactosaminidase	*Streptomyces xiaopingdaonensis*	90/93	WP_016910517
5	110	VOC family protein	*Streptomyces sp.* CT34	37/42	WP_043264817
6	190	hypothetical protein	*Streptomyces xiaopingdaonensis*	97/98	WP_033196419
7	327	4Fe-4S dicluster domain-containing protein	*Streptomyces xiaopingdaonensis*	96/97	WP_050988667
8	306	polysulfide reductase	*Streptomyces xiaopingdaonensis*	92/96	WP_016910522
9	188	formate dehydrogenase	*Streptomyces xiaopingdaonensis*	98/100	WP_237720310
10	886	formate dehydrogenase	*Streptomyces xiaopingdaonensis*	95/97	WP_237720310
11	349	selenide, water dikinase SelD	*Streptomyces xiaopingdaonensis*	97/97	WP_016910529
12	598	SelB C-terminal domain-containing protein	*Streptomyces xiaopingdaonensis*	95/97	WP_016910531
13	446	L-seryl-tRNA(Sec) selenium transferase	*Streptomyces xiaopingdaonensis*	95/96	WP_016910532
14	331	hypothetical protein	*Streptomyces diacarni*	72/80	RCG20951
15	228	HAD-IA family hydrolase	*Streptomyces xiaopingdaonensis*	86/92	WP_016906789
16	92	DUF4232 domain-containing protein	*Streptomyces purpurascens*	80/84	WP_267943032
17	1854	type I polyketide synthase	*Streptomyces armeniacus*	45/57	WP_208878173
18	1526	type I polyketide synthase	*Streptomyces sp.* TP-A0874	71/80	WP_069811168
19	2185	type I polyketide synthase	*Streptomyces sp.* TP-A0874	72/80	WP_069811170
20	1849	type I polyketide synthase	*Streptomyces sp.* TP-A0874	72/79	WP_069811172

genes	Size (AA)	Product	Source	Identity/ Positives	Accession Number
21	1497	type I polyketide synthase	*Streptomyces sp.* TP-A0874	70/77	WP_079127094
22	1731	condensation domain-containing protein	*Streptomyces sp.* TP-A0874	70/78	WP_069811173
23	272	thioesterase	*Streptomyces sp.* SID3343	66/75	WP_237535452
24	533	acyl-CoA carboxylase subunit beta	*Streptomyces sp.* TP-A0874	85/92	WP_069811177
25	89	acyl-CoA carboxylase subunit epsilon	*Streptomyces sp.* TP-A0874	54/70	WP_069811179
26	586	fatty acyl-AMP ligase	*Streptomyces sp.* SID3343	69/78	WP_161358761
27	681	amino acid adenylation domain-containing protein	*Streptomyces sp.* TP-A0874	72/81	WP_244501035
28	594	biotin carboxylase N-terminal domain-containing protein	*Streptomyces cacaoi*	91/95	WP_086816657
29	525	DHA2 family efflux MFS transporter permease subunit	*Streptomyces sp.* TP-A0874	76/84	WP_069811182
30	194	TetR family transcriptional regulator	*Streptomyces sp.* TP-A0874	78/91	WP_170837721
31	194	TetR family transcriptional regulator	*Streptomyces hoynatensis*	74/81	WP_241562282
32	161	Lrp/AsnC family transcriptional regulator	*Sinosporangium siamense*	48/68	WP_204027989
33	108	Lrp/AsnC family transcriptional regulator	*Streptomyces apricus*	51/70	WP_149513023
34	537	gamma-glutamyltransferase	*Streptomyces iconiensis*	92/95	WP_274042445
35	446	MFS transporter	*Streptomyces iconiensis*	87/91	WP_274042446
36	551	hydantoinase B/oxoprolinase family protein	*Streptomyces iconiensis*	94/96	WP_274042447

Figure S1. The molecular network obtained by combining the LC-MS/MS analyses of six fractions of extracts from strain DSM 40104.

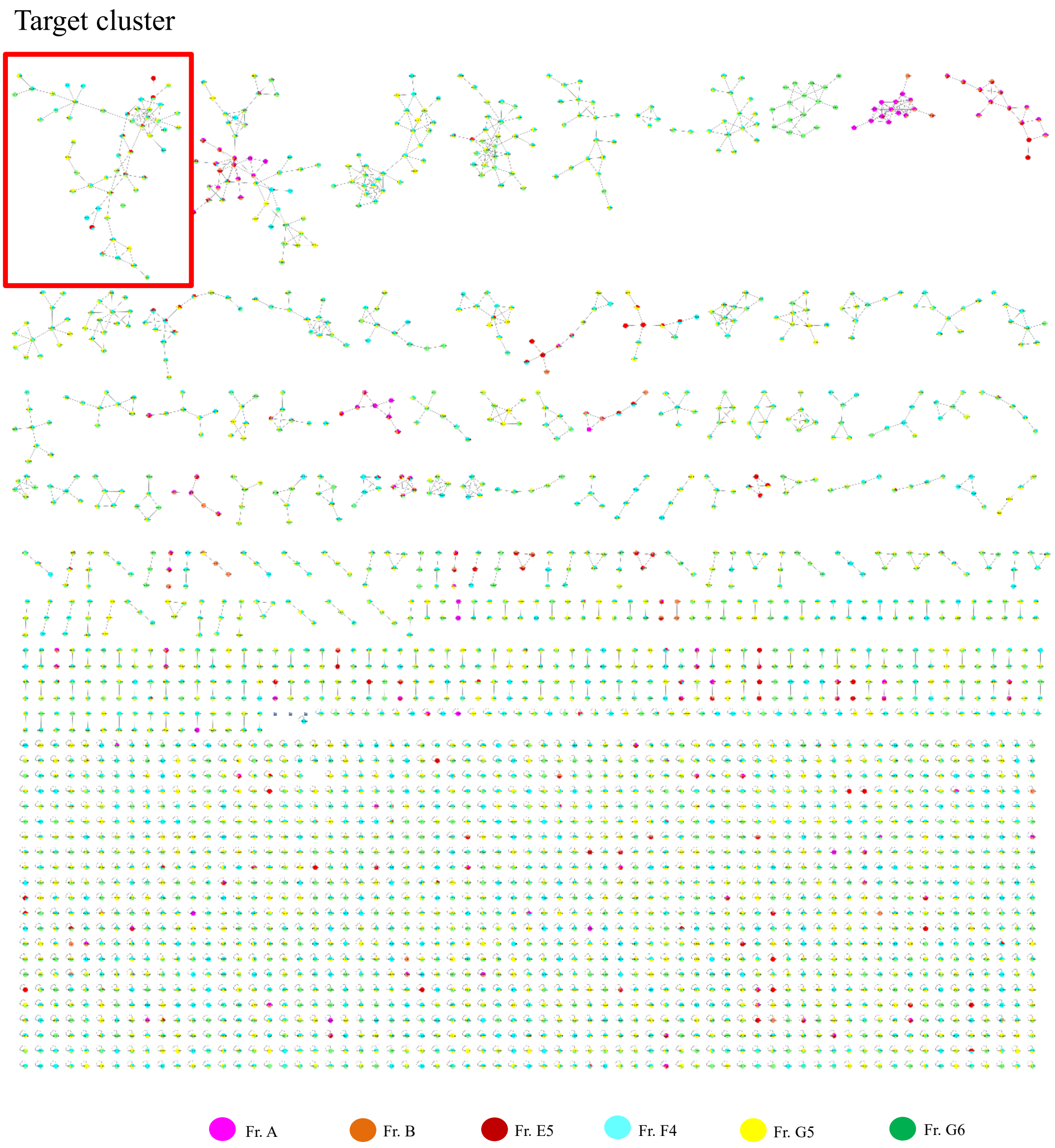

Figure S2. Target cluster extract from the molecular network

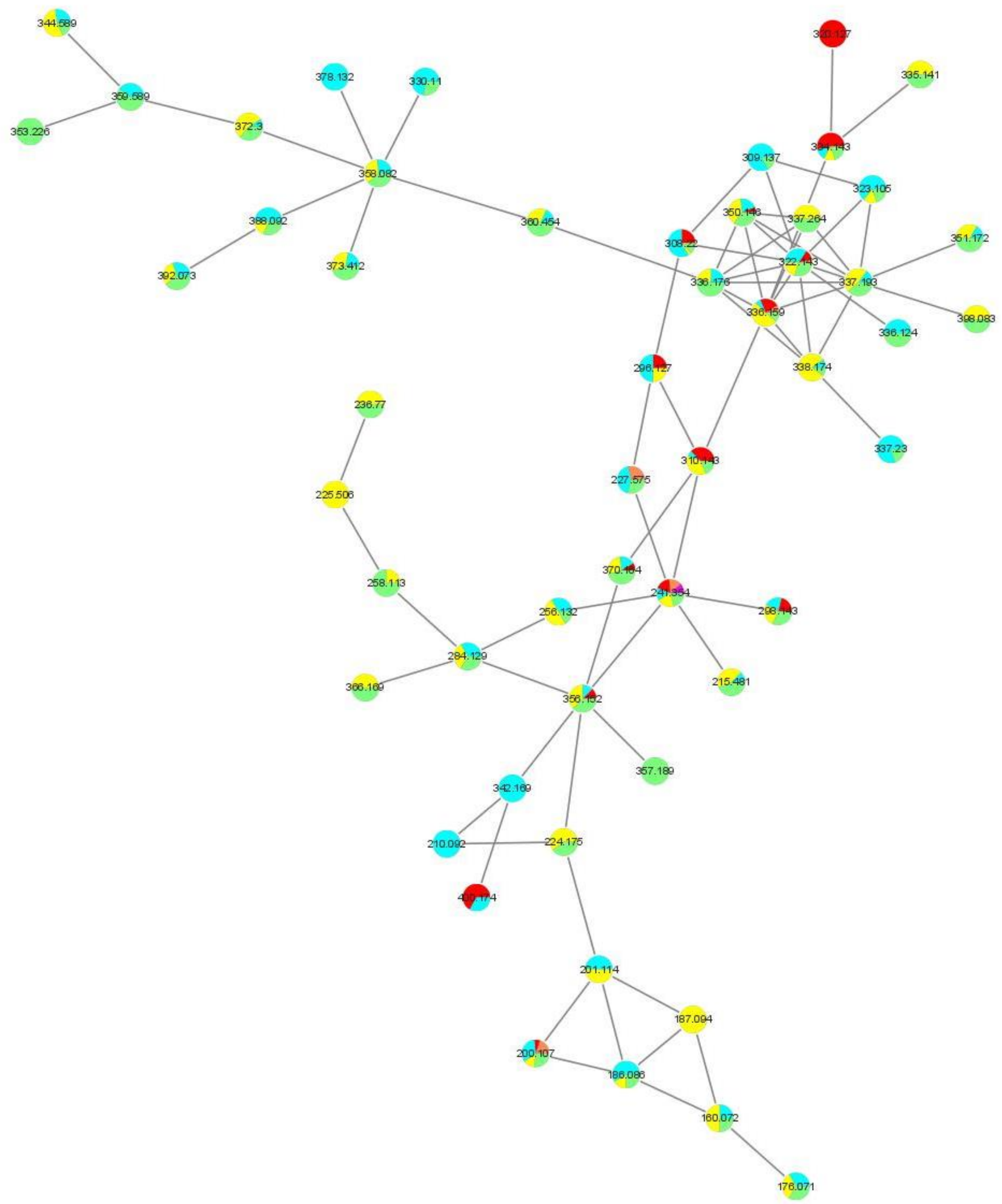

Figure S3. The alignment result of all KR domains in the *pyi* gene cluster

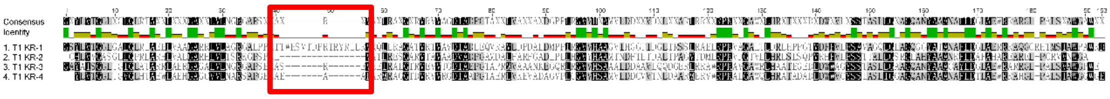

Figure S4. The alignment result of both DH domains in the *pyi* gene cluster

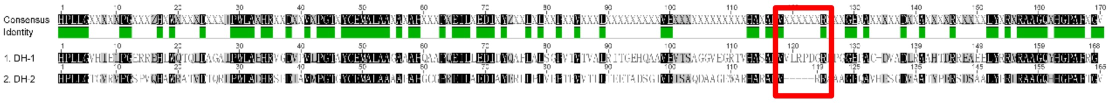

Figure S5. The conserved domains analysis of A domains in the *pyi* gene cluster

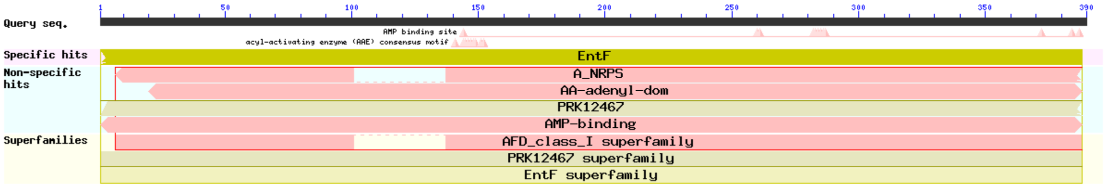

Figure S6. Inhibitory activity of compounds **1~3** (50 μM) on NO production in LPS-induced BV-2 cells

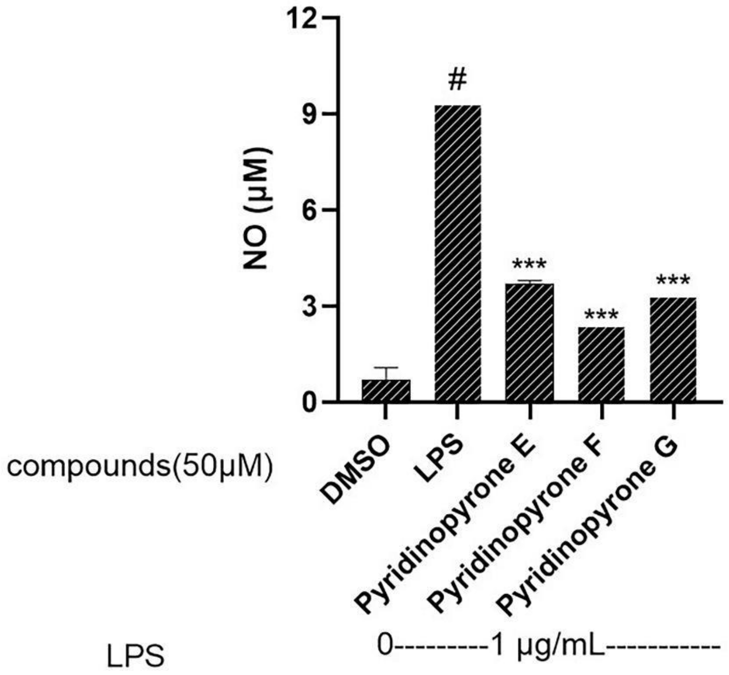

Figure S7. Inhibitory activity of dexamethasone with different concentrations on NO production in LPS-induced BV-2 cells

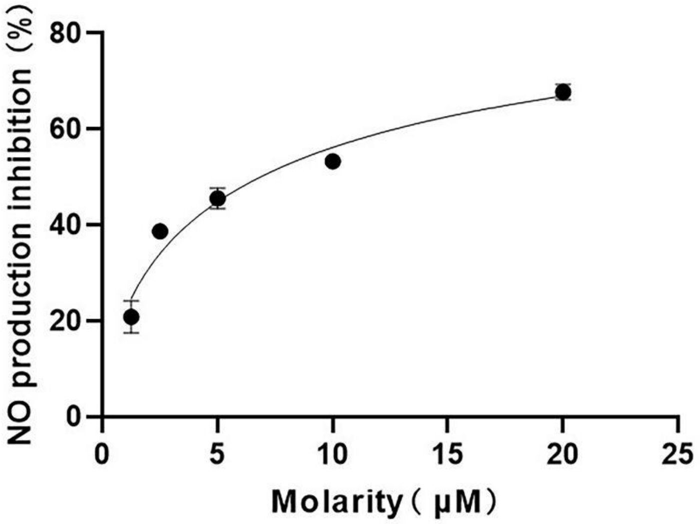

Figure S8. ^{1}H NMR spectrum of pyridinopyrone E (**1**) (400 MHz, DMSO-d_6)

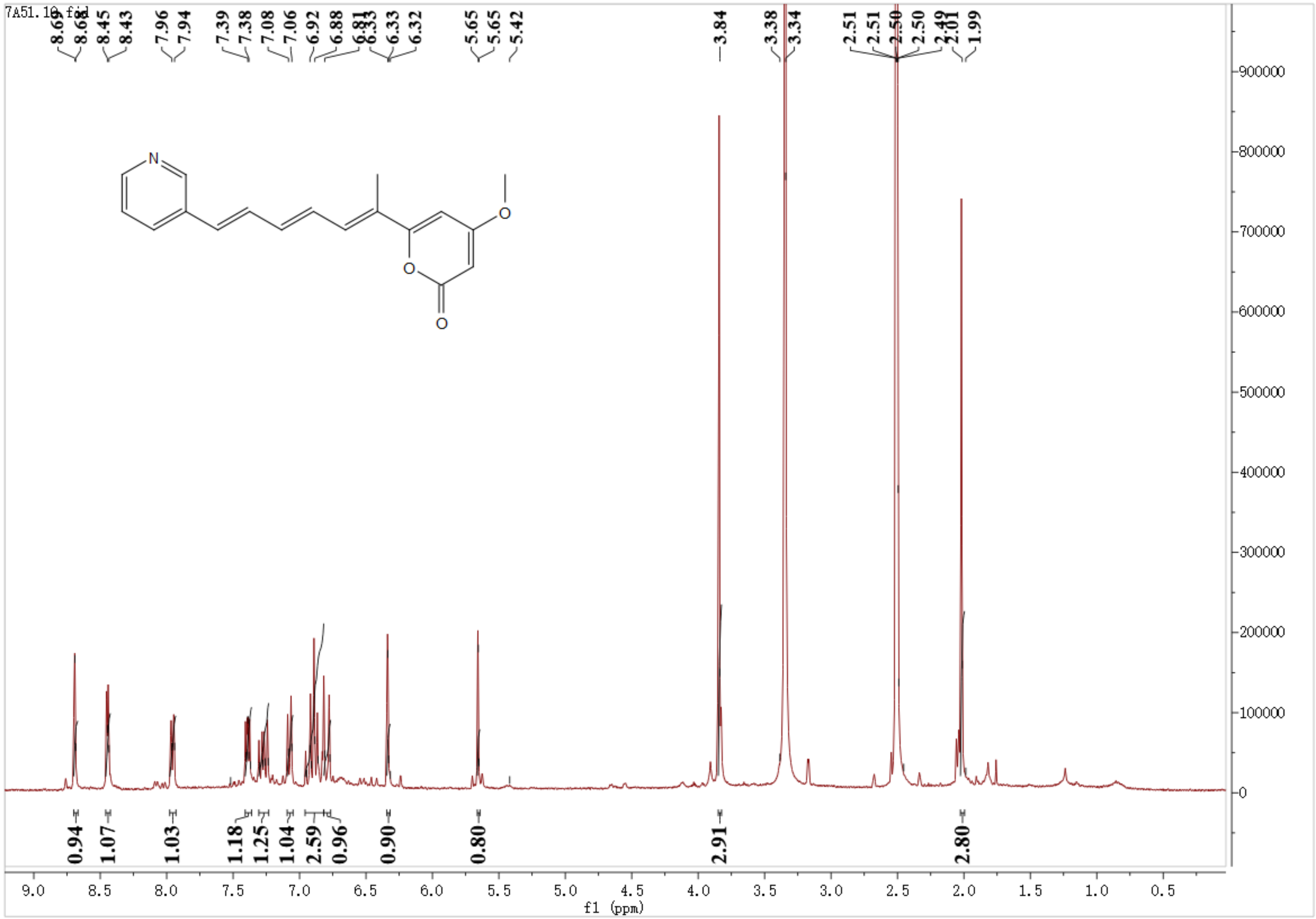

Figure S9. ^{13}C NMR spectrum of pyridinopyrone E (**1**) (400 MHz, DMSO-*d*$_6$)

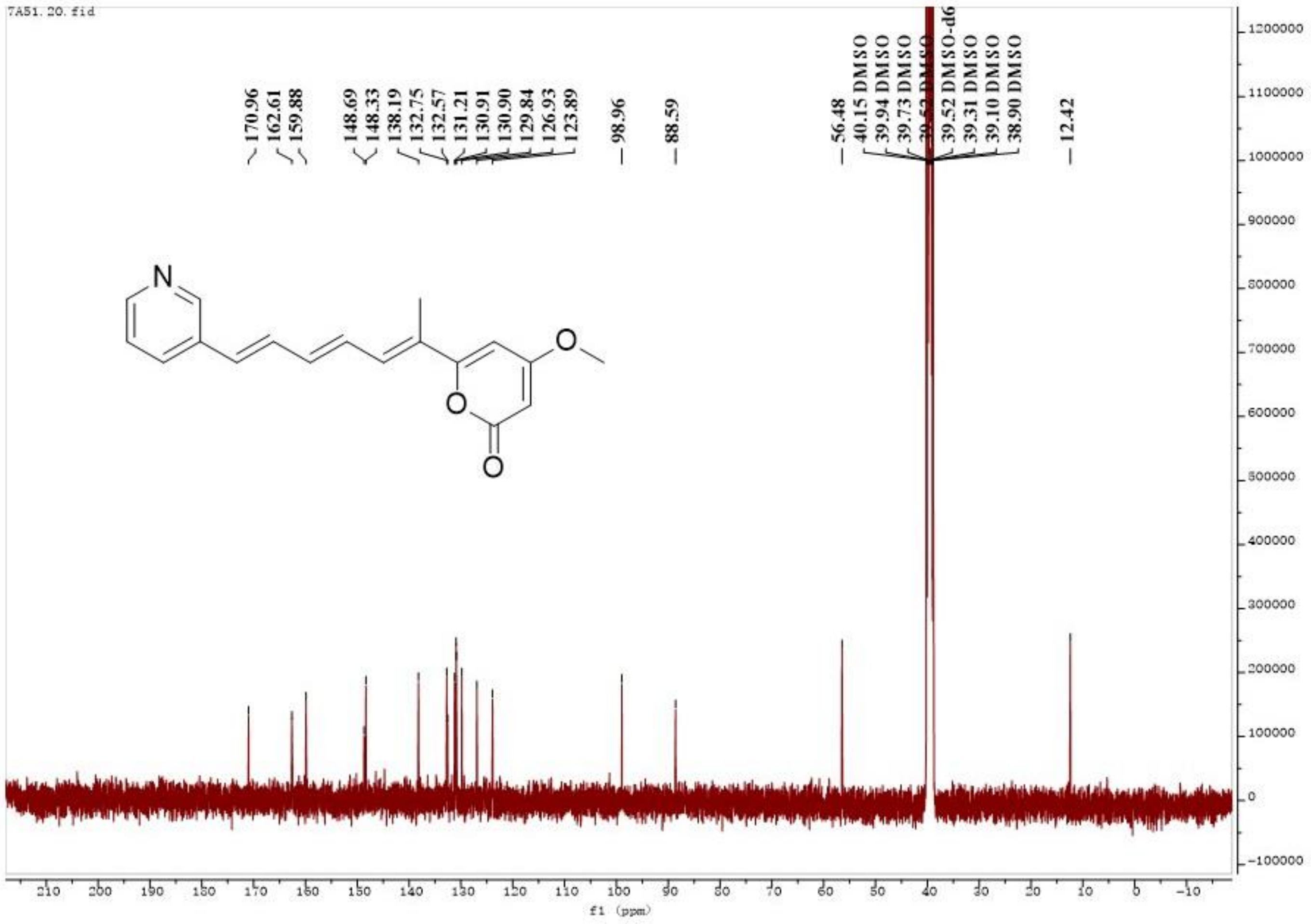

Figure S10. HSQC spectrum of pyridinopyrone E (**1**) (DMSO-d_6)

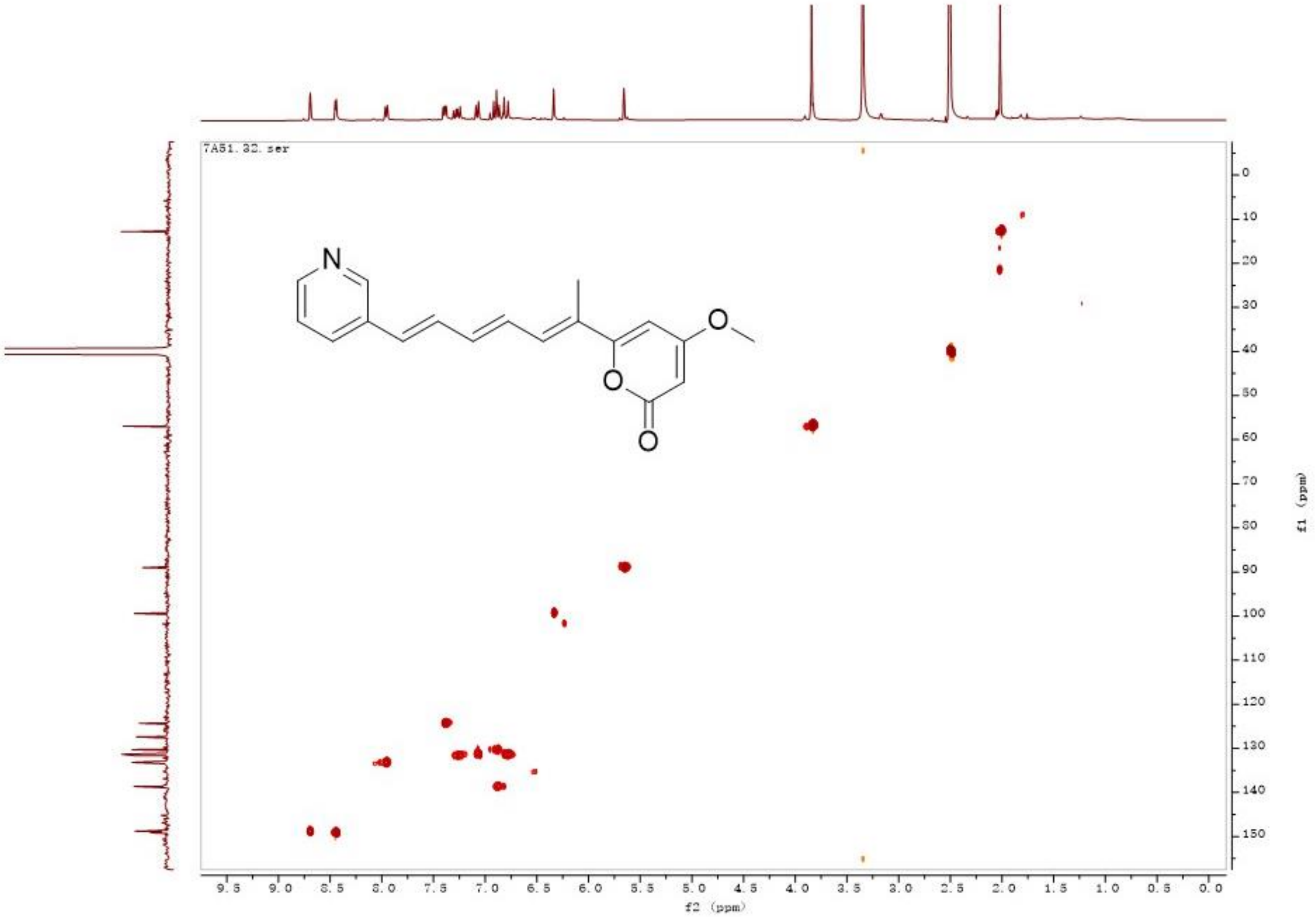

Figure S11. ^{1}H-^{1}H COSY spectrum of pyridinopyrone E (**1**) (DMSO-d_6)

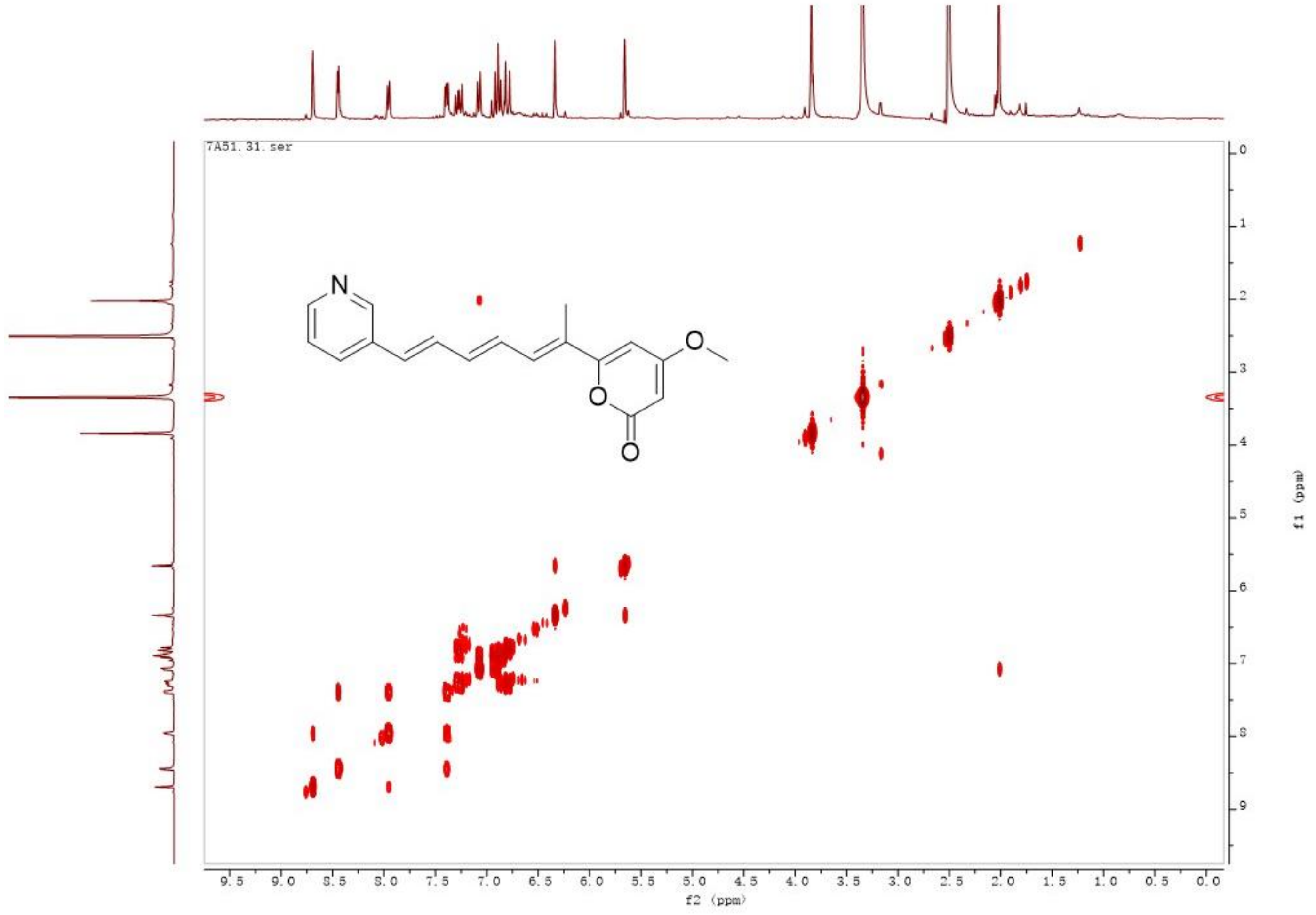

Figure S12. HMBC spectrum of pyridinopyrone E (**1**) (DMSO-d_6)

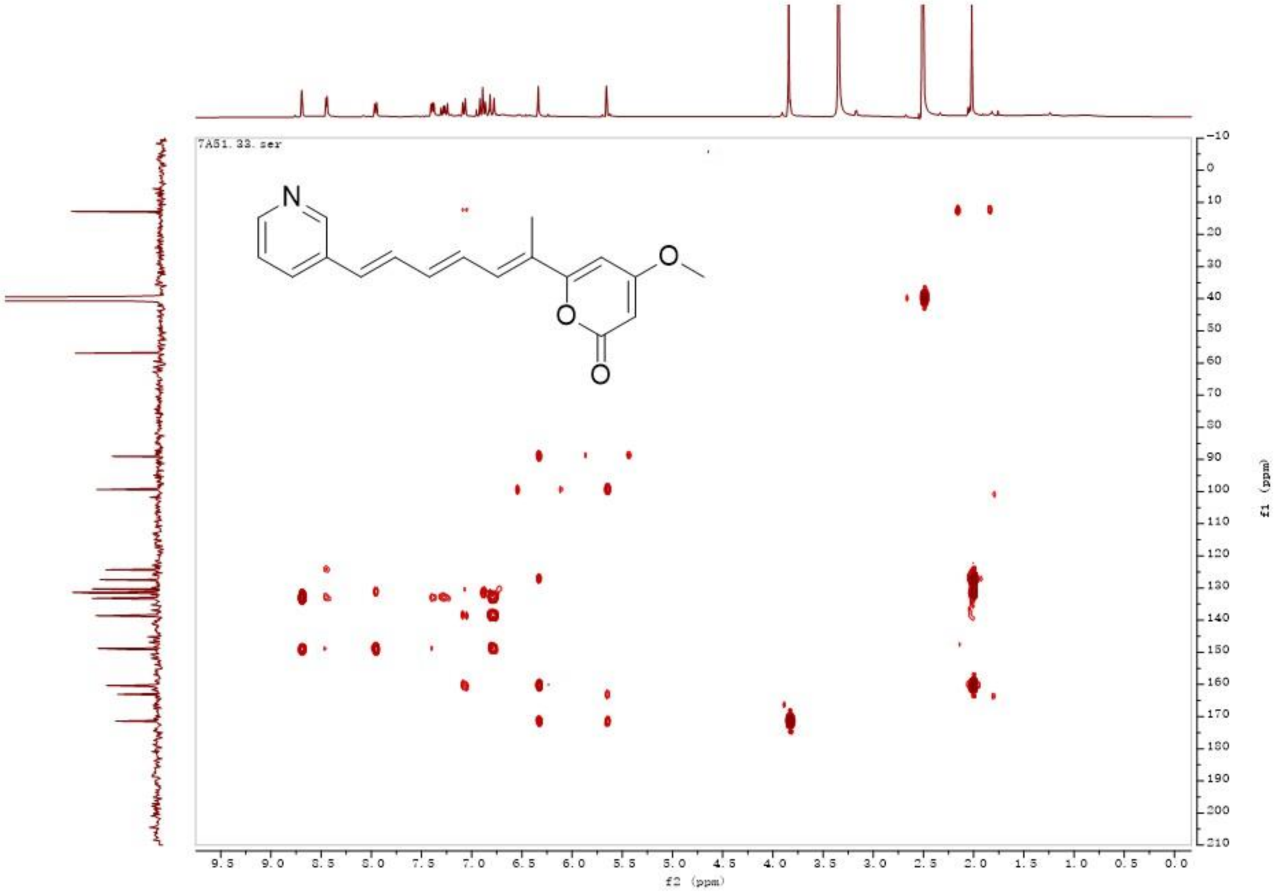

Figure S13. HRESIMS spectrum of pyridinopyrone E (**1**)

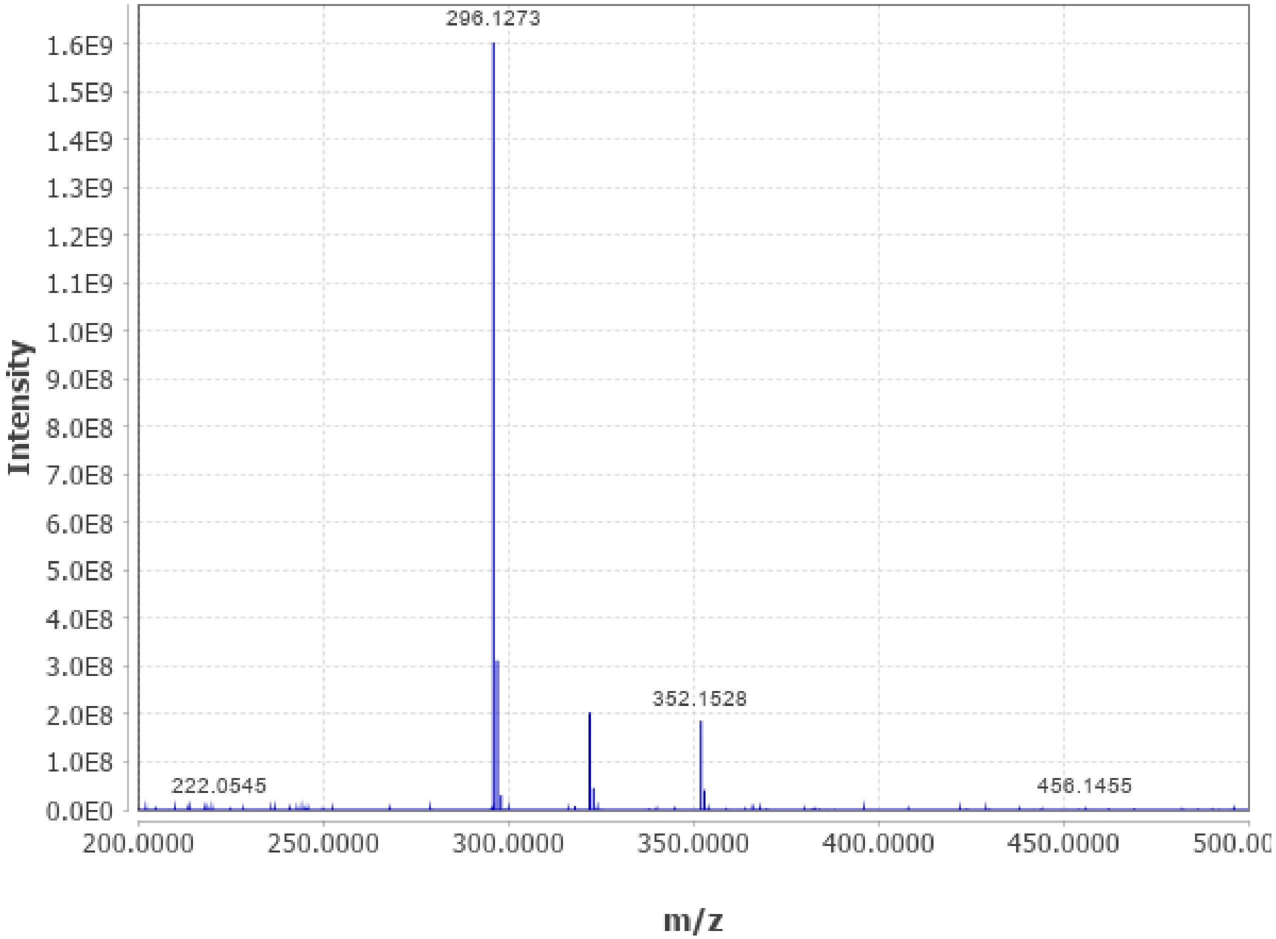

Figure S14. UV spectrum of pyridinopyrone E (**1**)

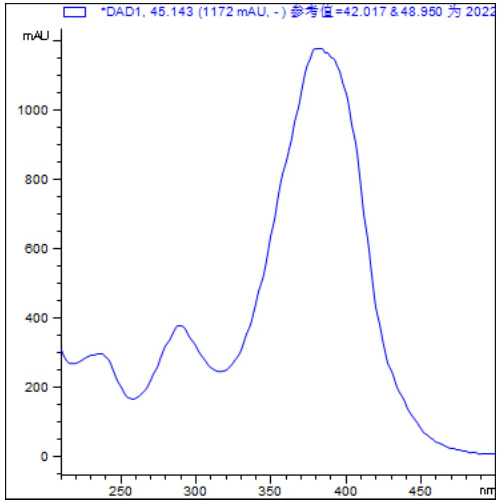

Figure S15. ^{1}H NMR spectrum of pyridinopyrone F (**2**) (400 MHz, DMSO-d_6)

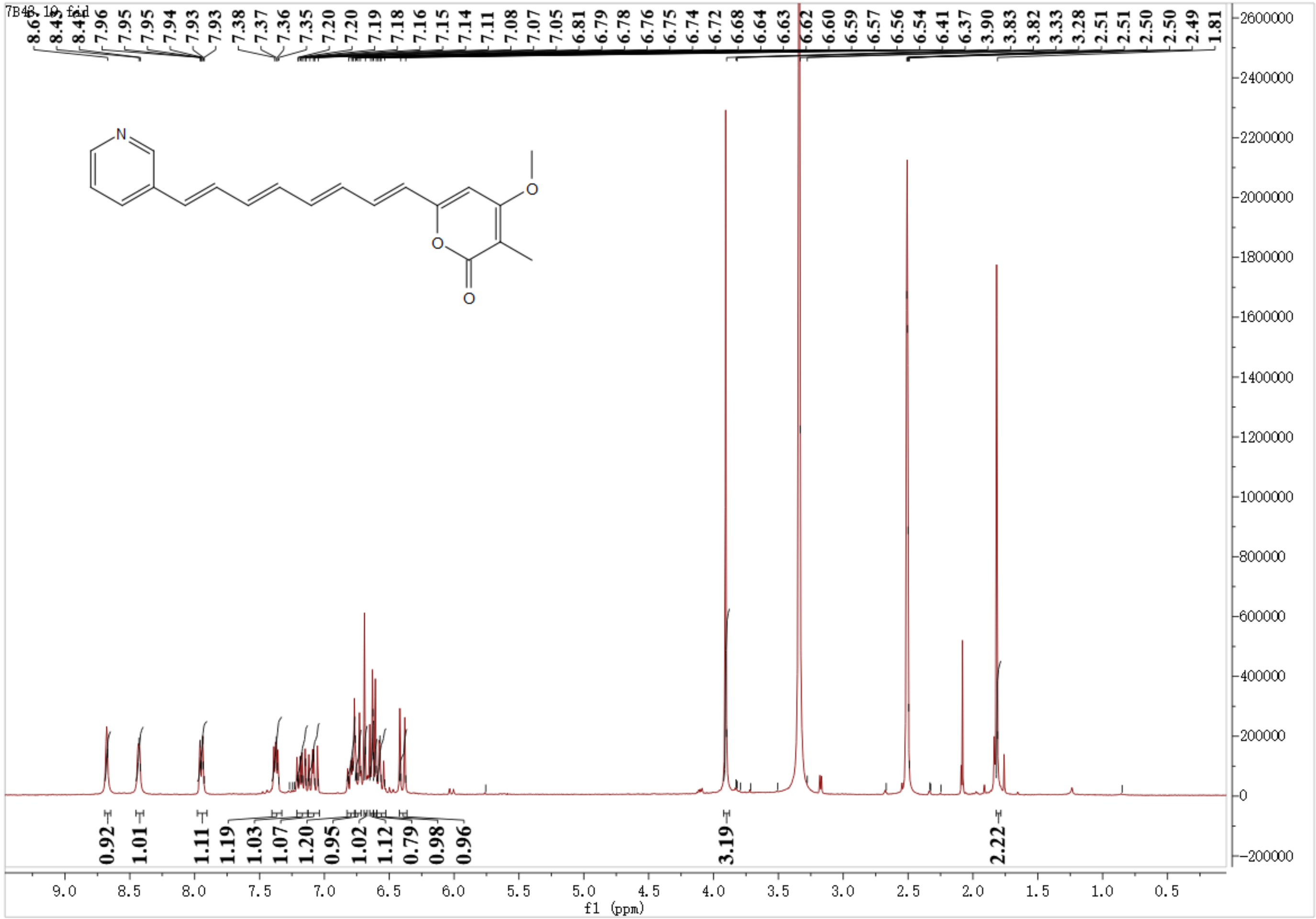

Figure S16. ^{13}C NMR spectrum of pyridinopyrone F (**2**) (400 MHz, DMSO-d_6)

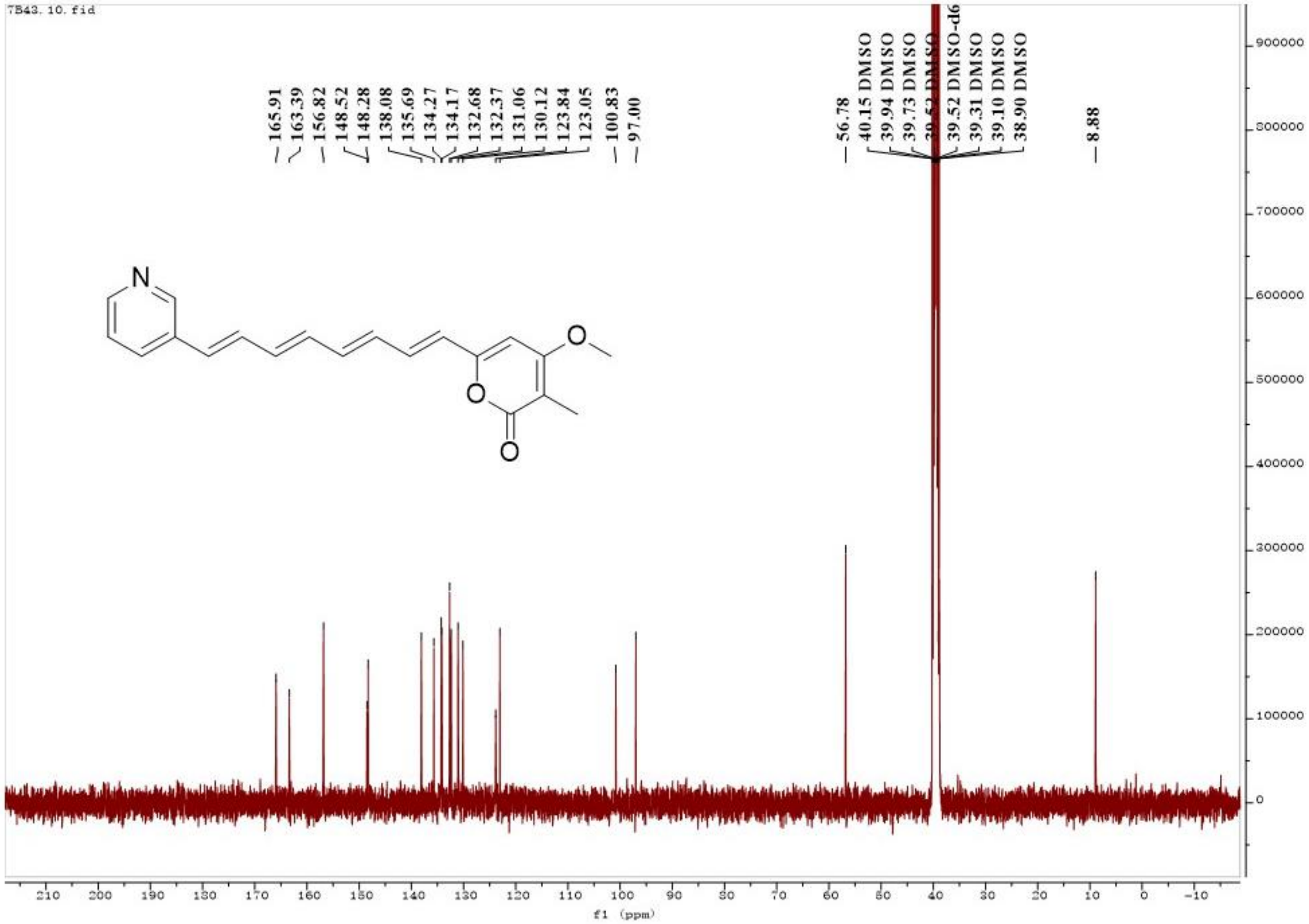

Figure S17. HSQC spectrum of pyridinopyrone F (**2**) (DMSO-d_6)

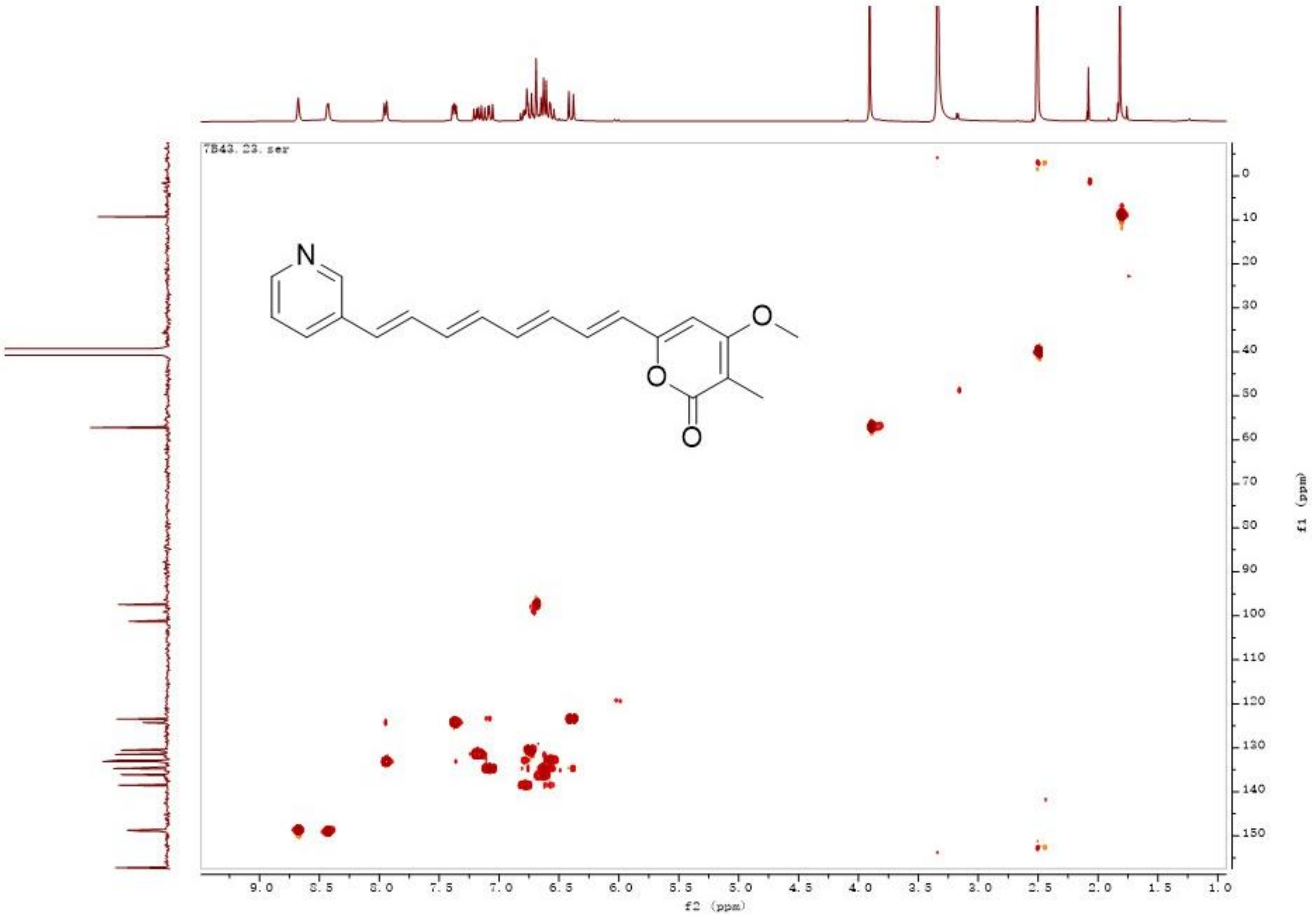

Figure S18. ^1H-^1H COSY spectrum of pyridinopyrone F (**2**) (DMSO-d_6)

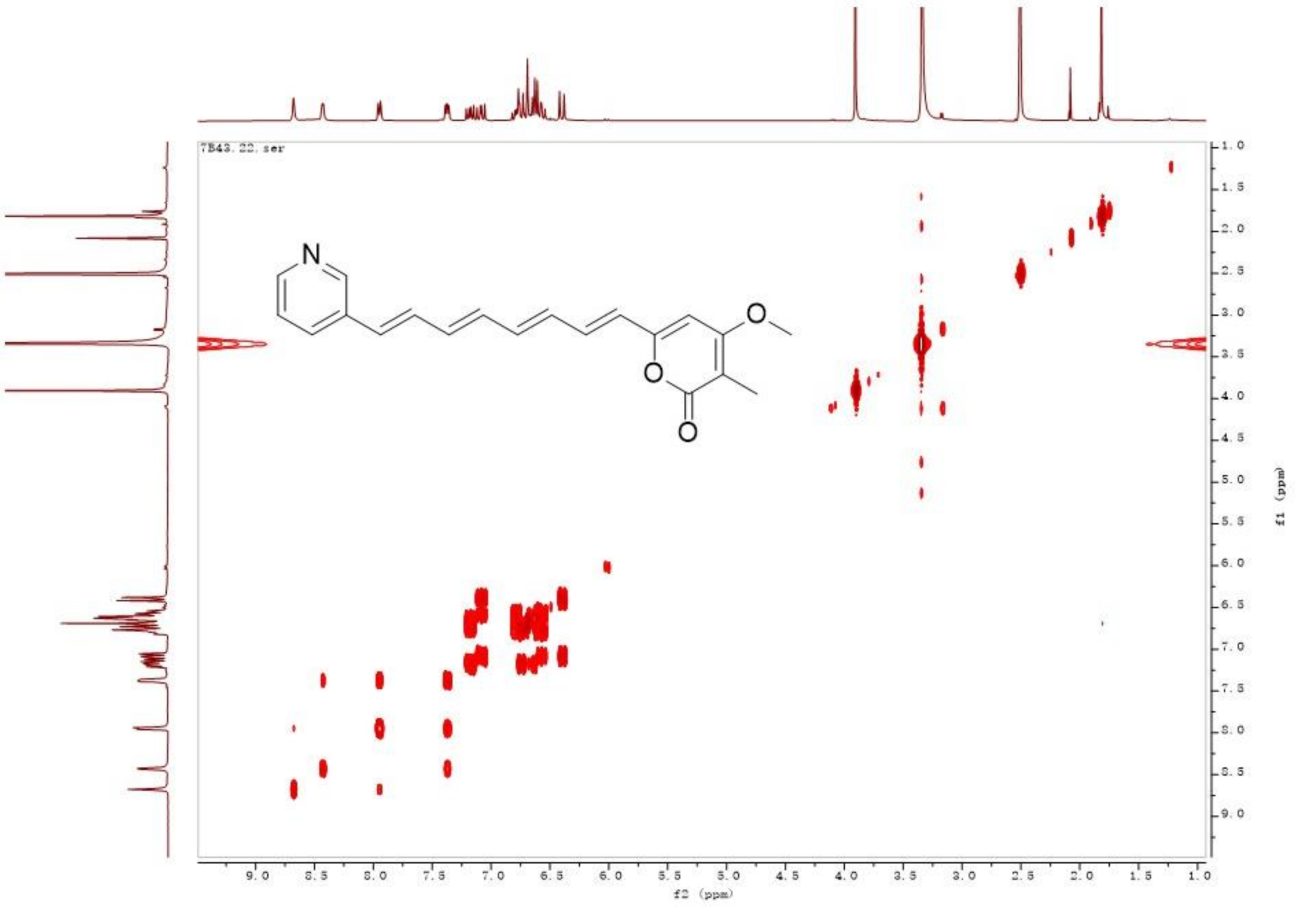

Figure S19. HMBC spectrum of pyridinopyrone F (**2**) (DMSO-d_6)

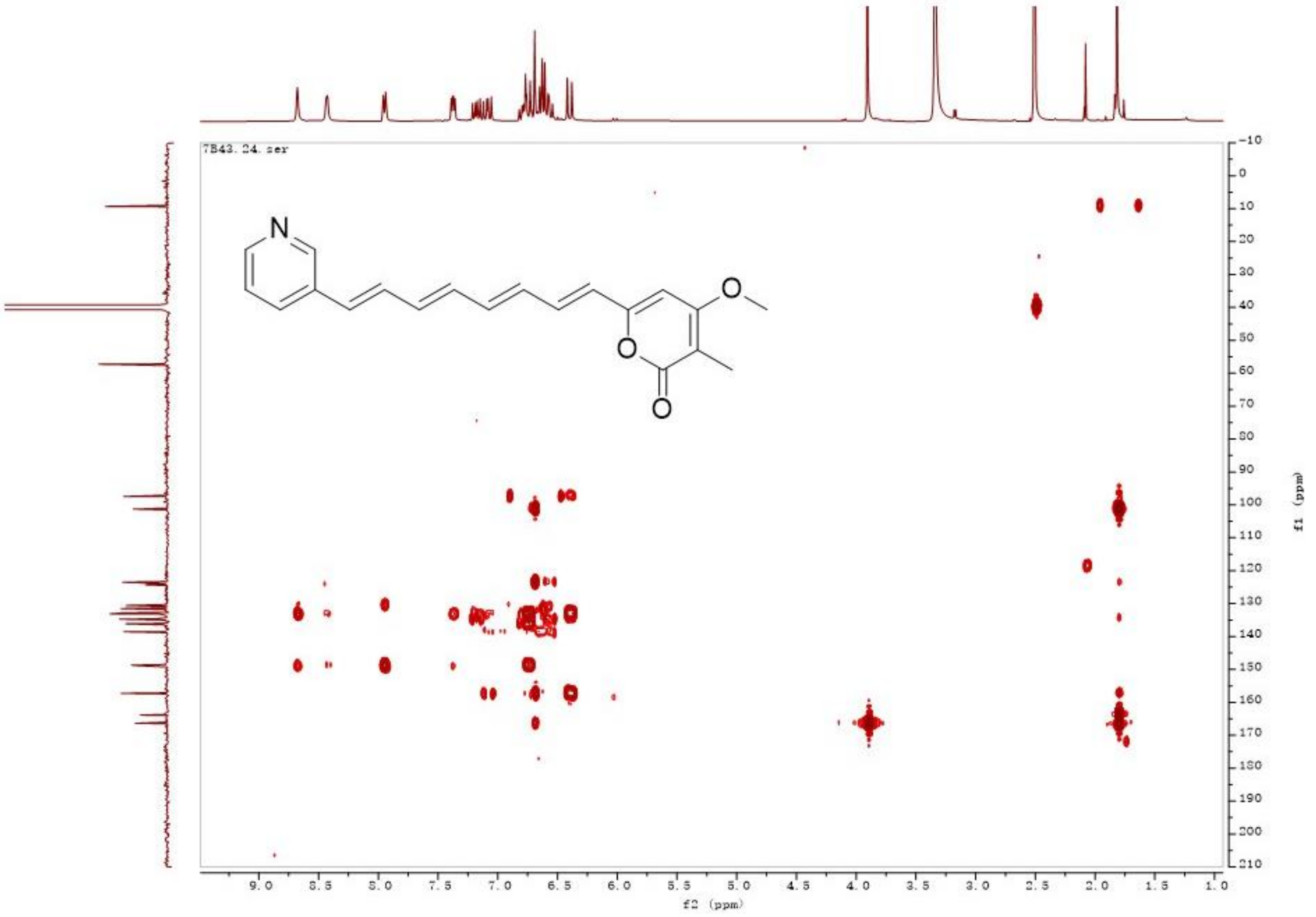

Figure S20. HRESIMS spectrum of pyridinopyrone F (**2**)

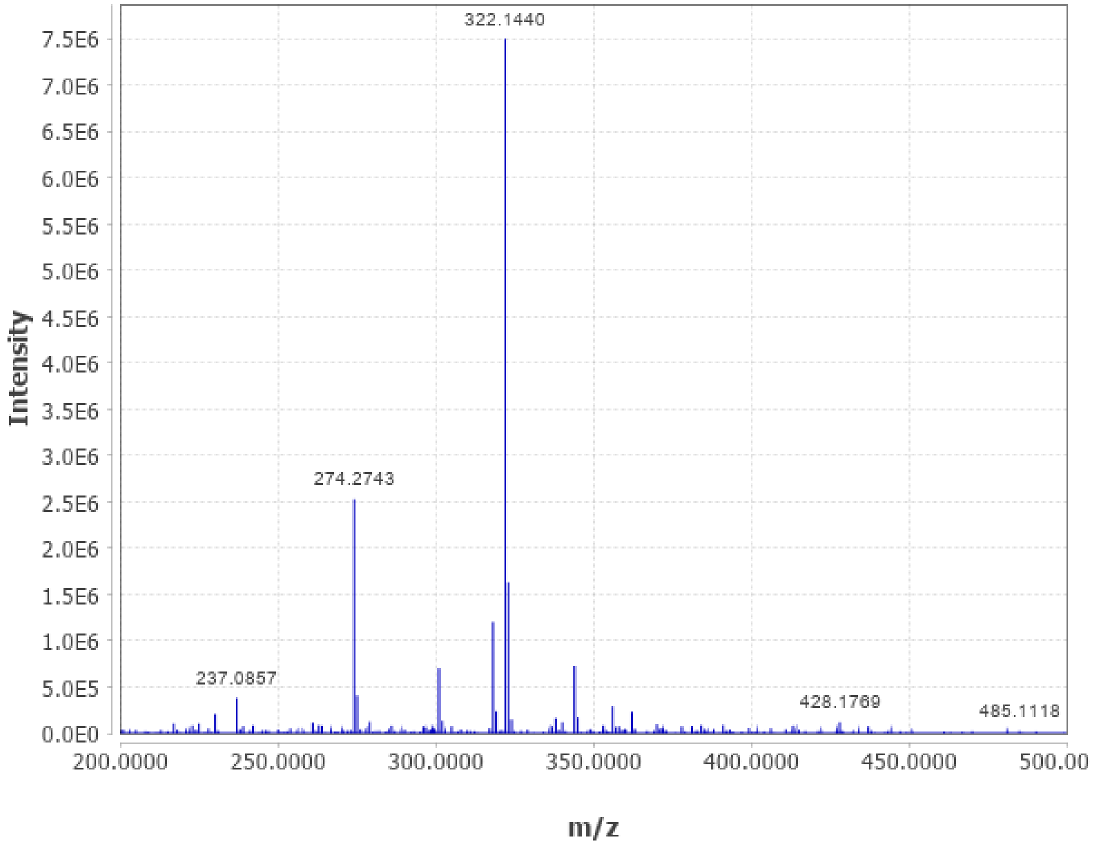

Figure S21. UV spectrum of pyridinopyrone F (**2**)

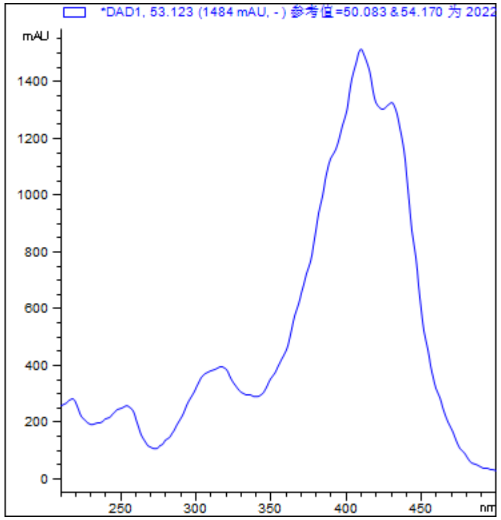

Figure S22. ^{1}H NMR spectrum of pyridinopyrone G (**3**) (400 MHz, DMSO-d_6)

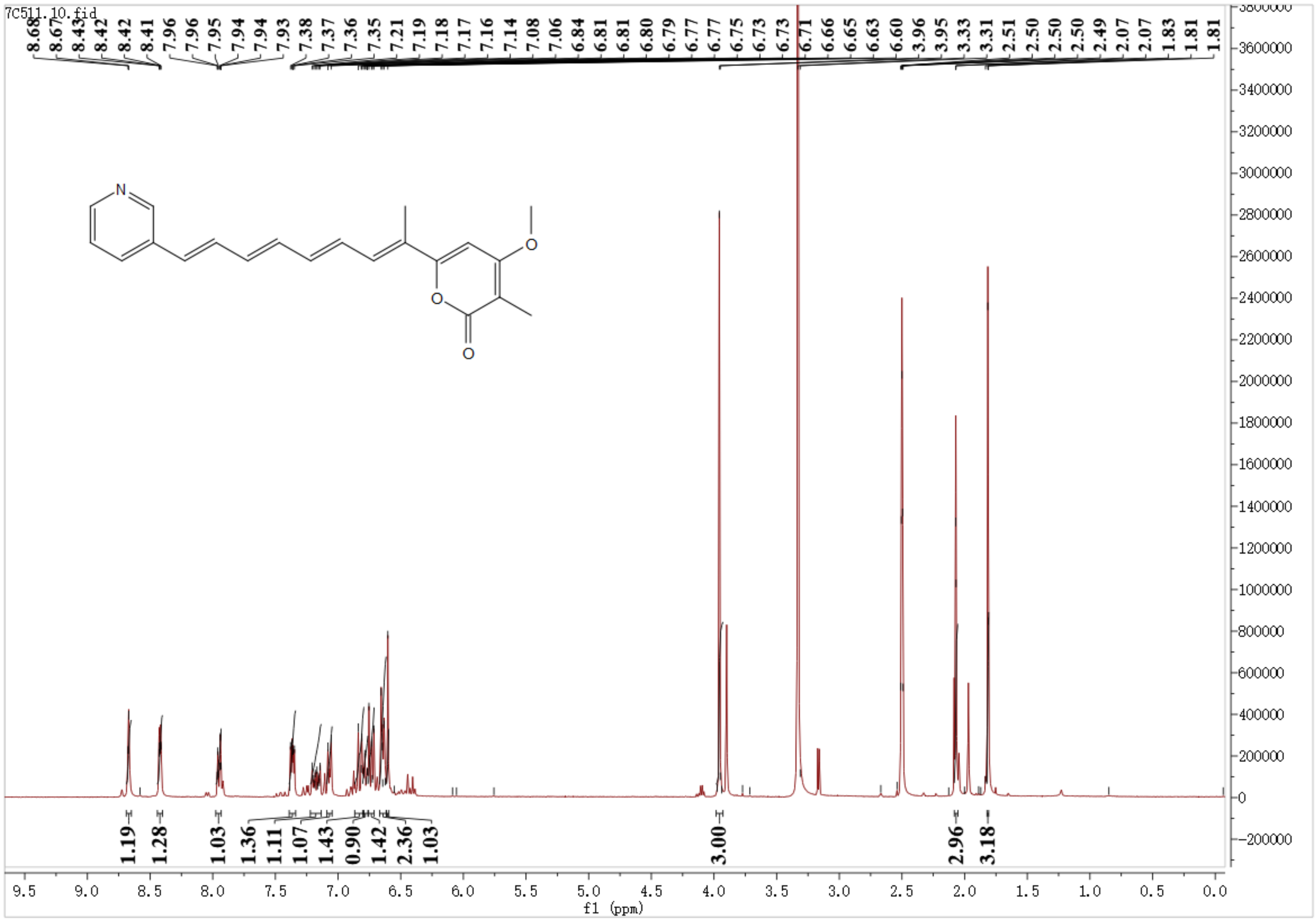

Figure S23. ^{13}C NMR spectrum of pyridinopyrone G (**3**) (400 MHz, DMSO-d_6)

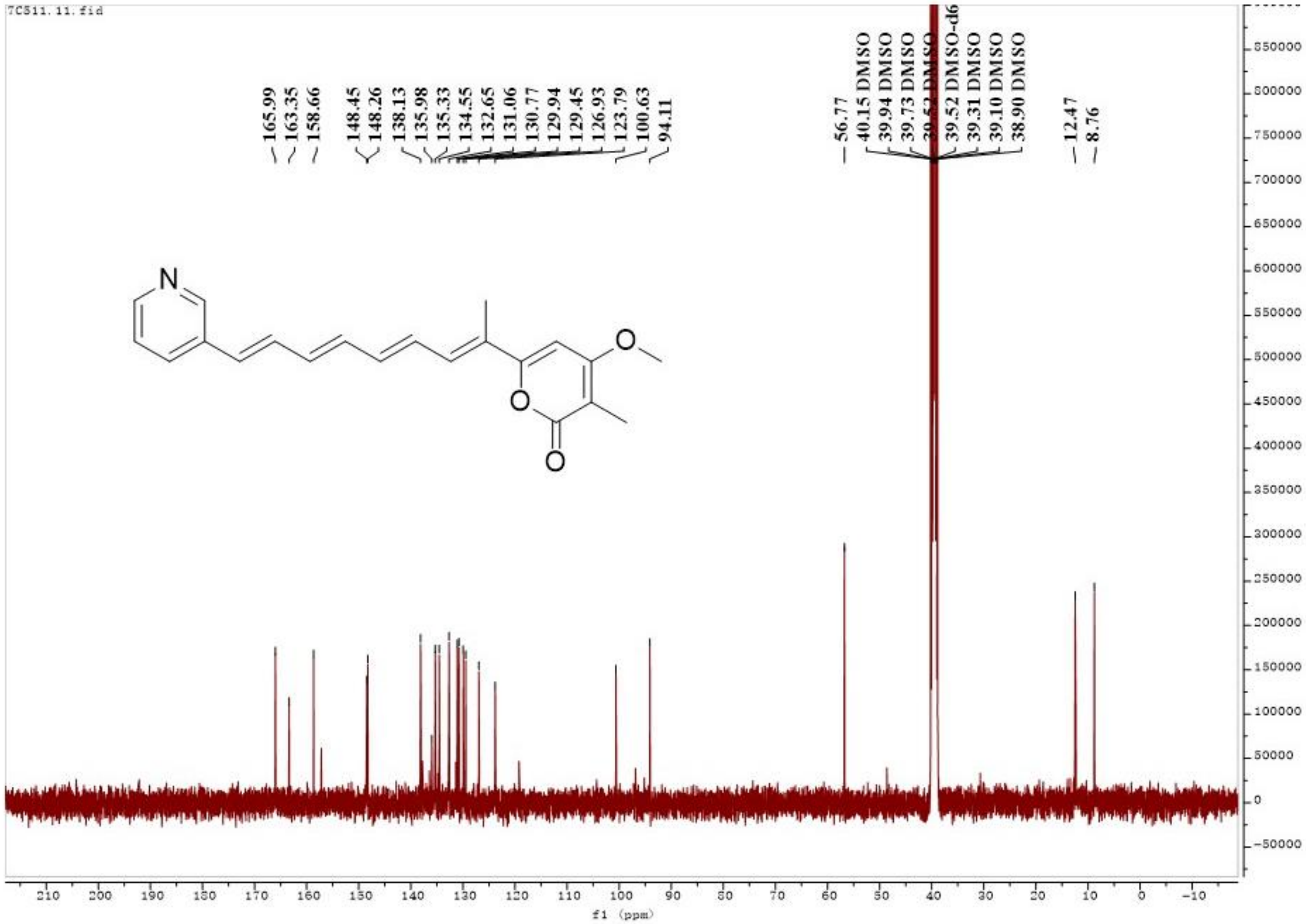

Figure S24. ^{1}H-^{1}H COSY spectrum of pyridinopyrone G (**3**) (DMSO-d_6)

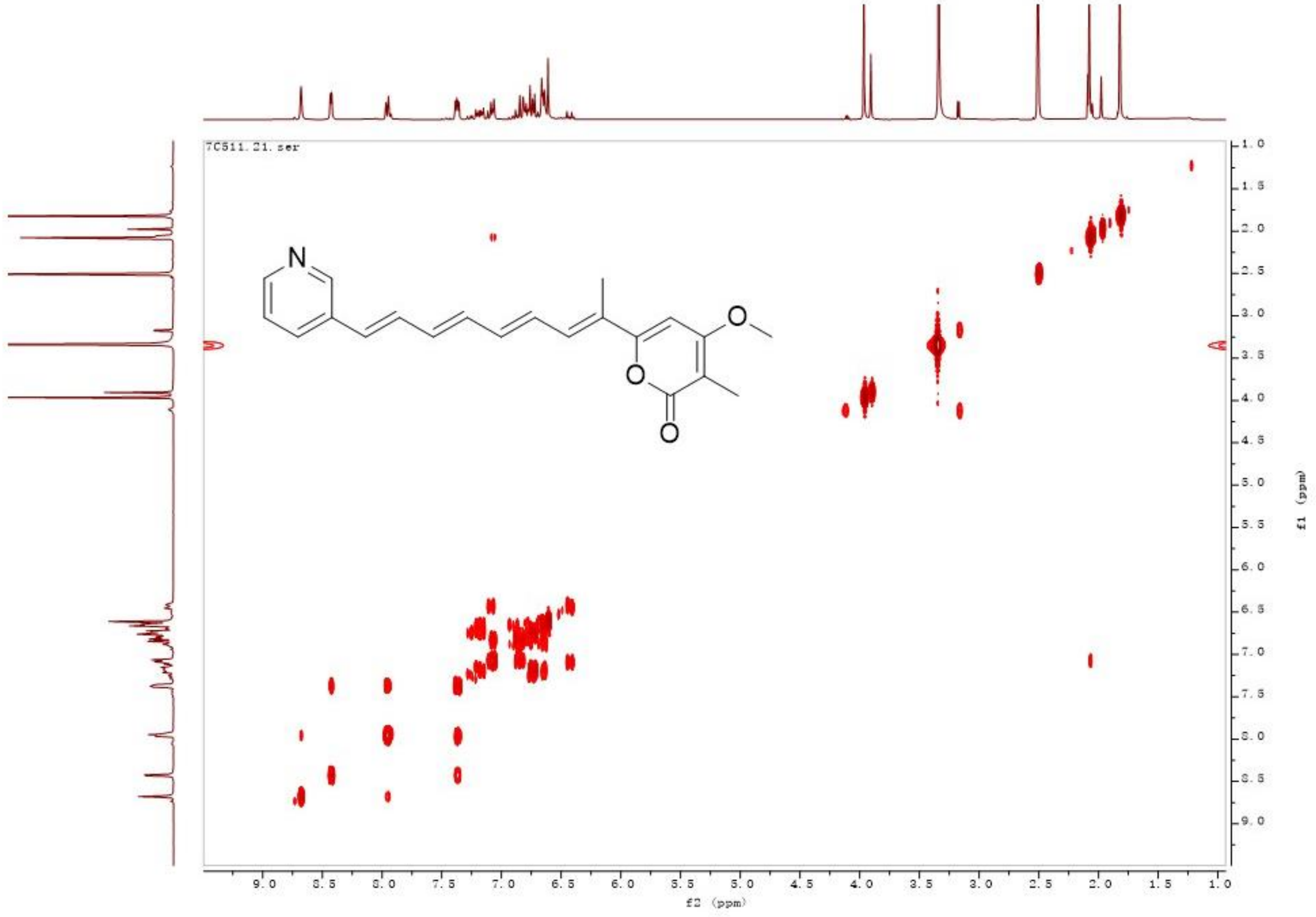

Figure S25. HSQC spectrum of pyridinopyrone G (**3**) (DMSO-d_6)

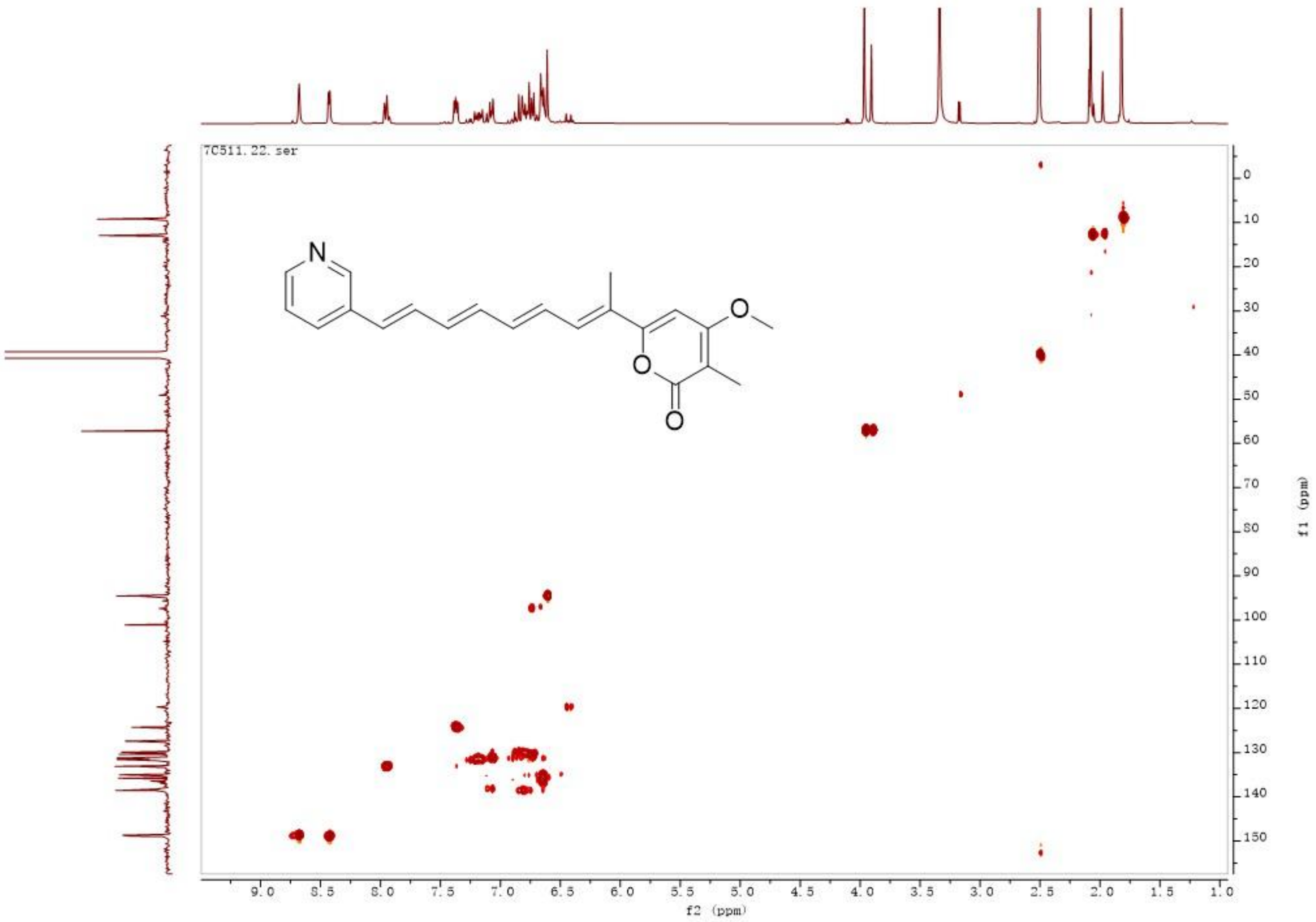

Figure S26. HMBC spectrum of pyridinopyrone G (**3**) (DMSO-d_6)

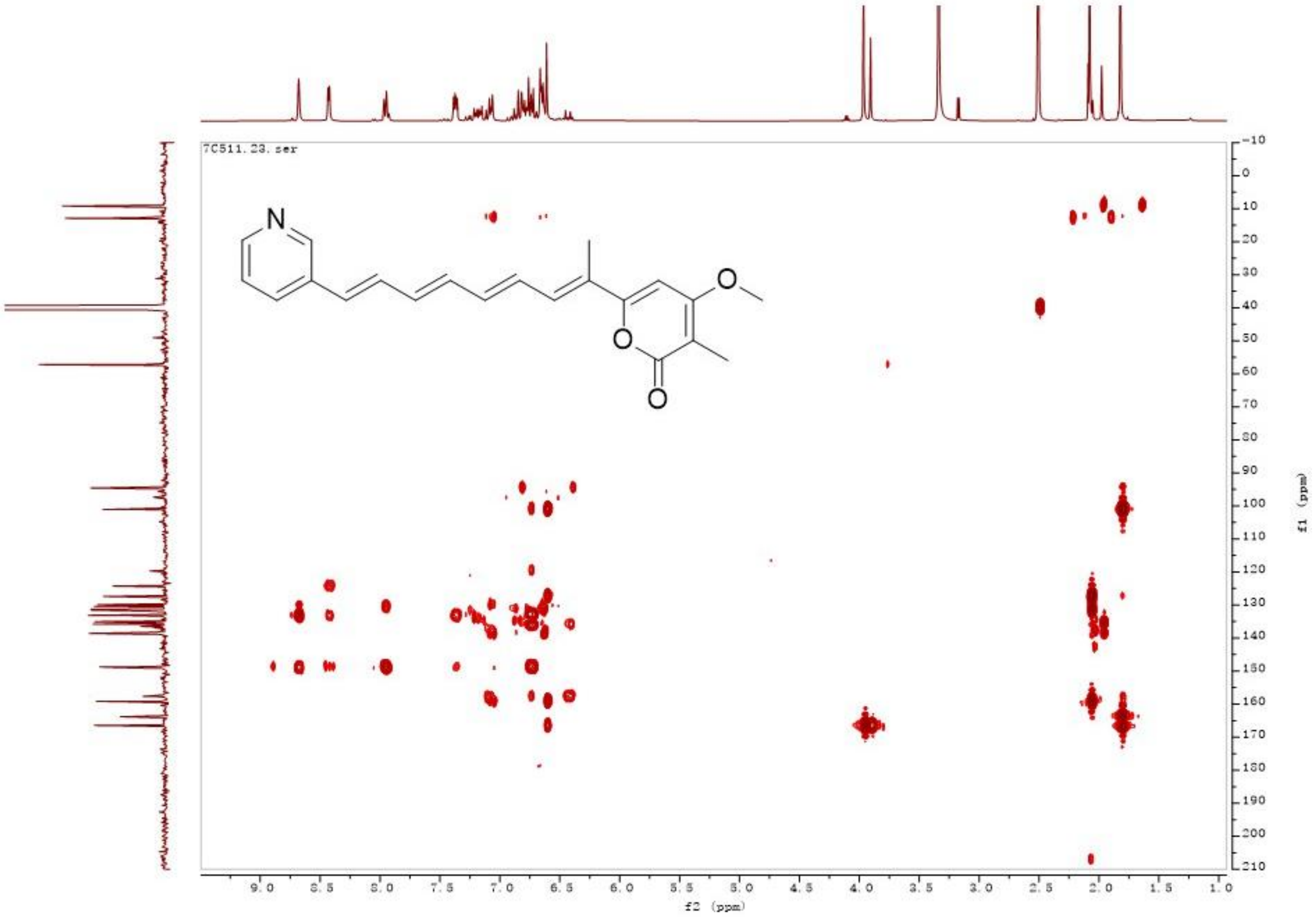

Figure S27. HRESIMS spectrum of pyridinopyrone G (**3**)

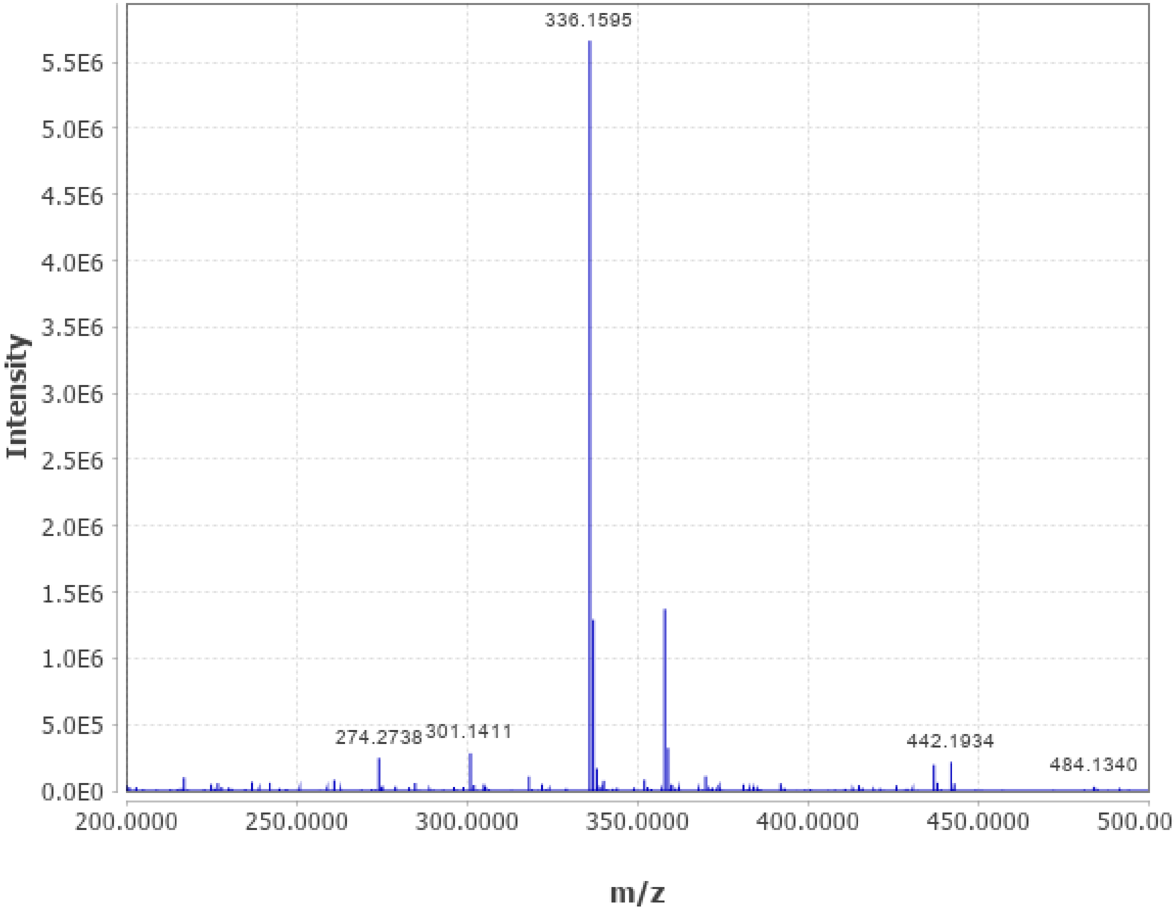

Figure S28. UV spectrum of pyridinopyrone G (**3**)

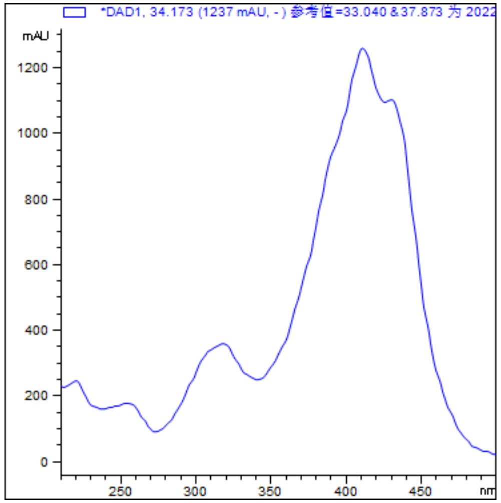

Figure S29. ^{1}H NMR spectrum of pyridinopyrone H (**4**) (400 MHz, DMSO-d_6)

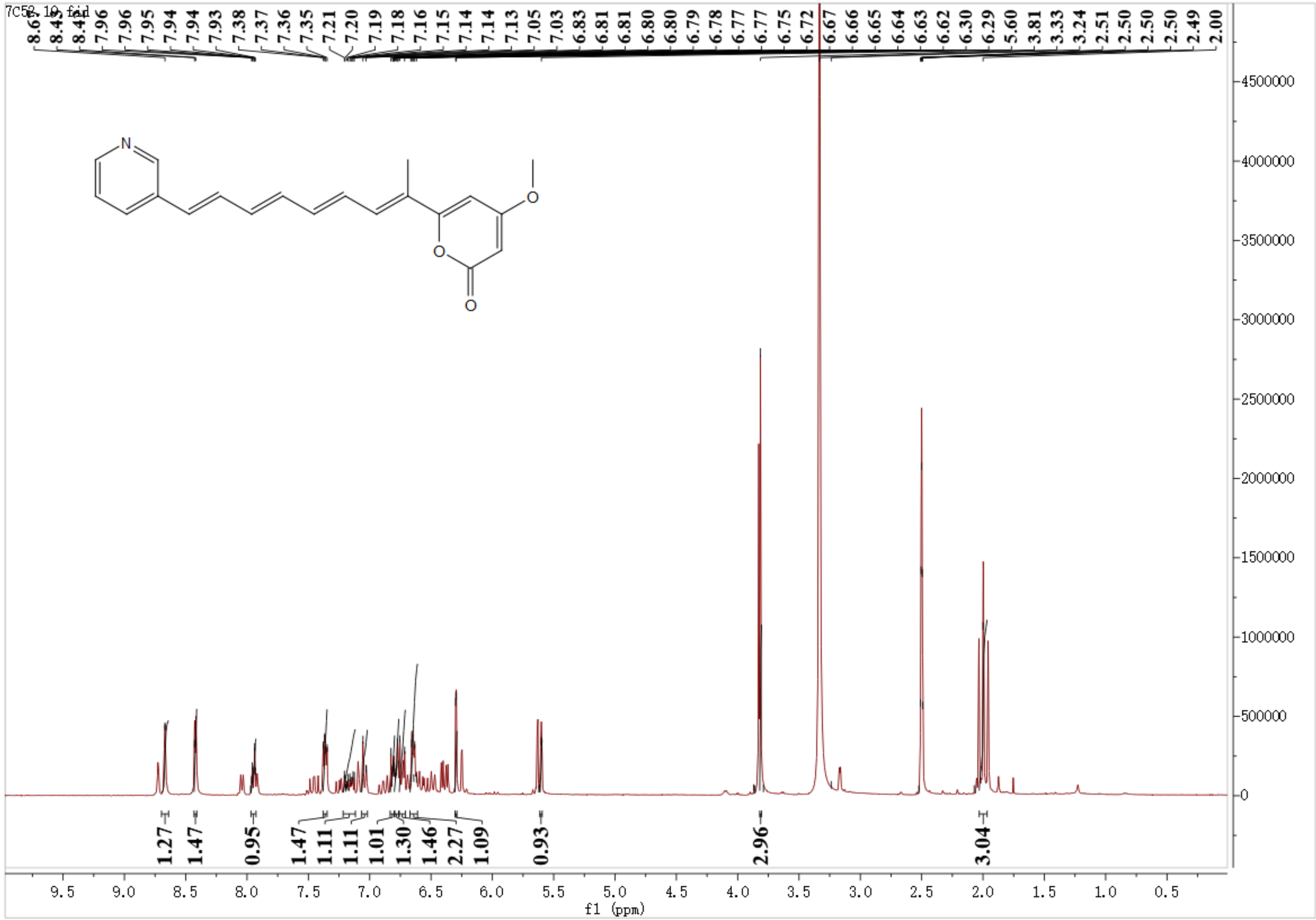

Figure S30. ^{13}C NMR spectrum of pyridinopyrone H (**4**) (400 MHz, DMSO-d_6)

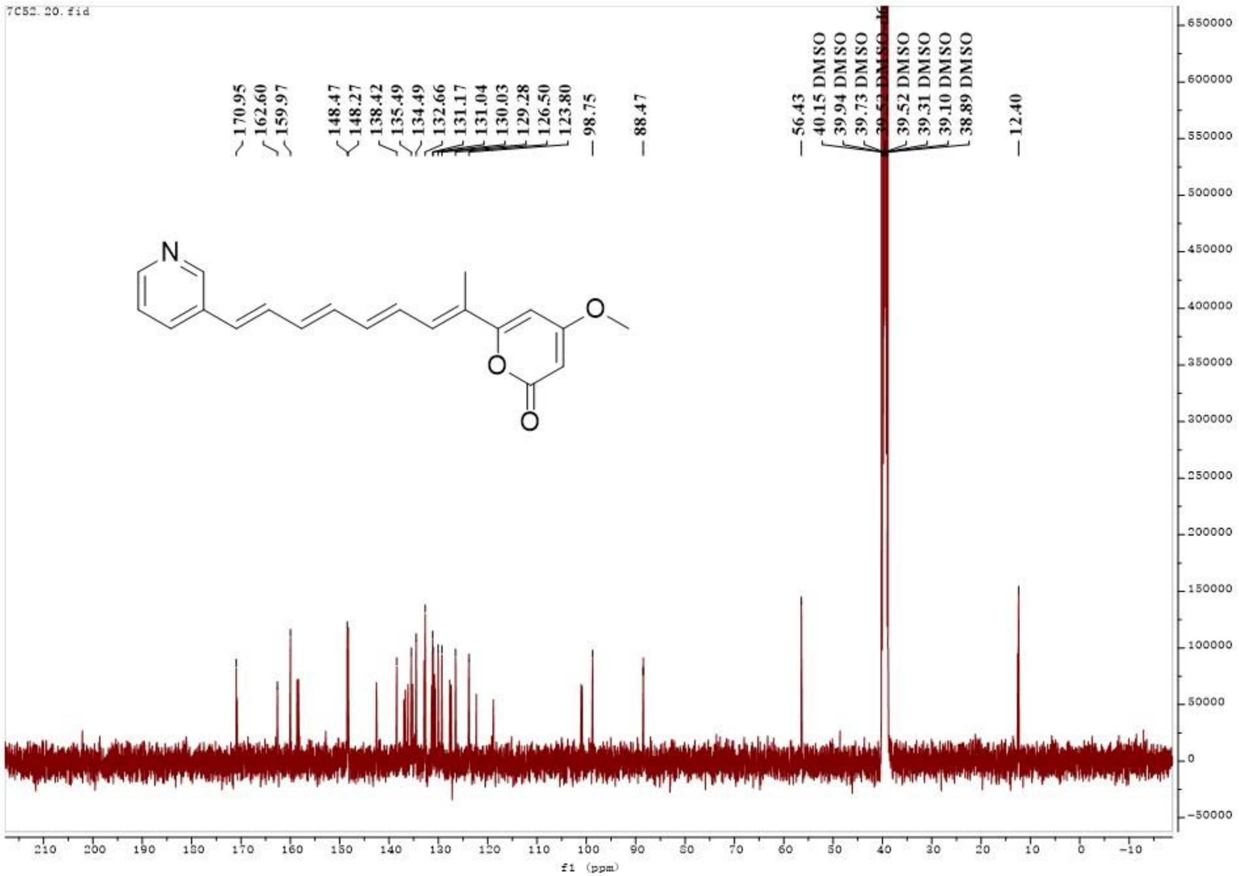

Figure S31. ^1H-^1H COSY spectrum of pyridinopyrone H (**4**) (DMSO-d_6)

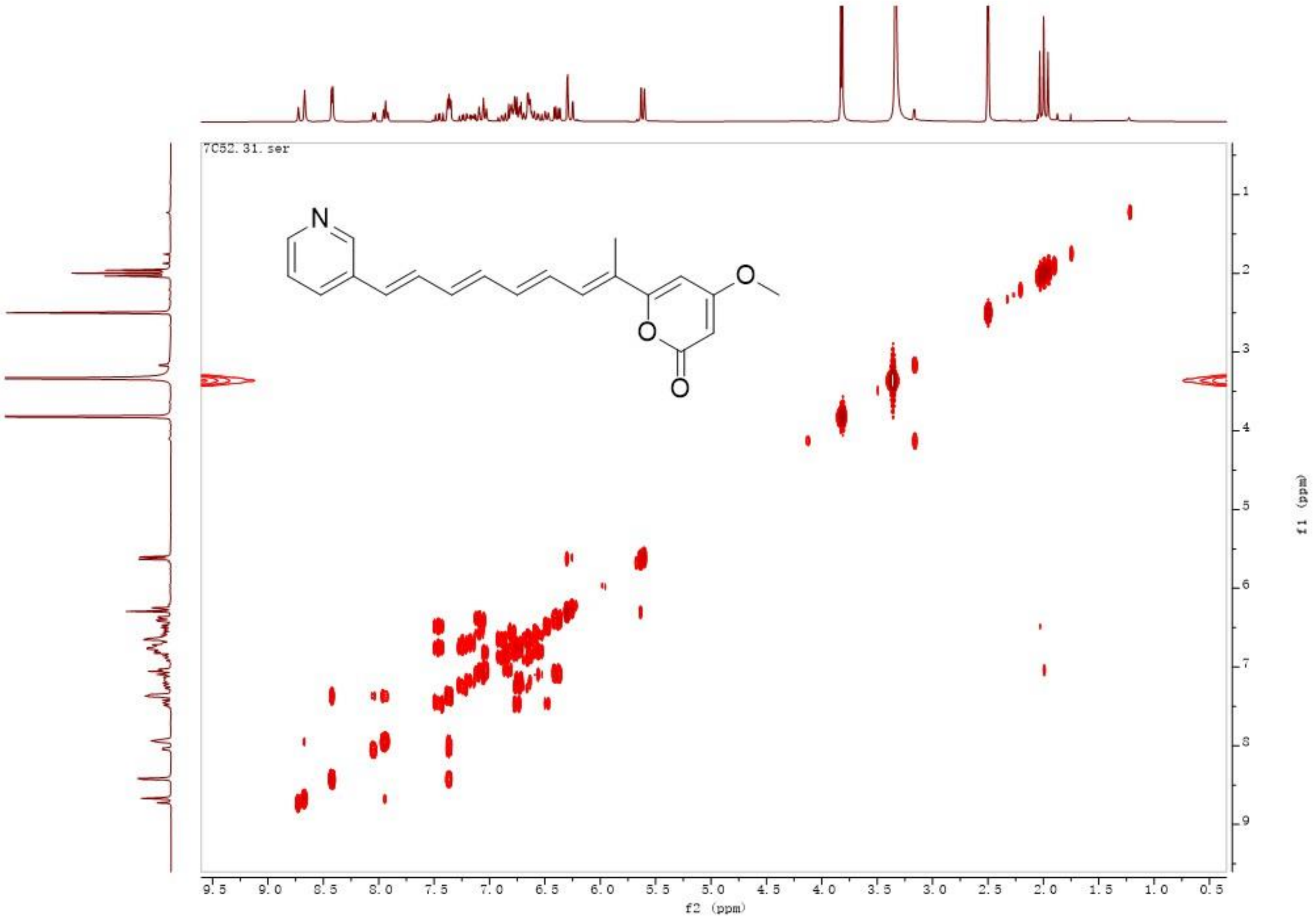

Figure S32. HSQC spectrum of pyridinopyrone H (**4**) (DMSO-d_6)

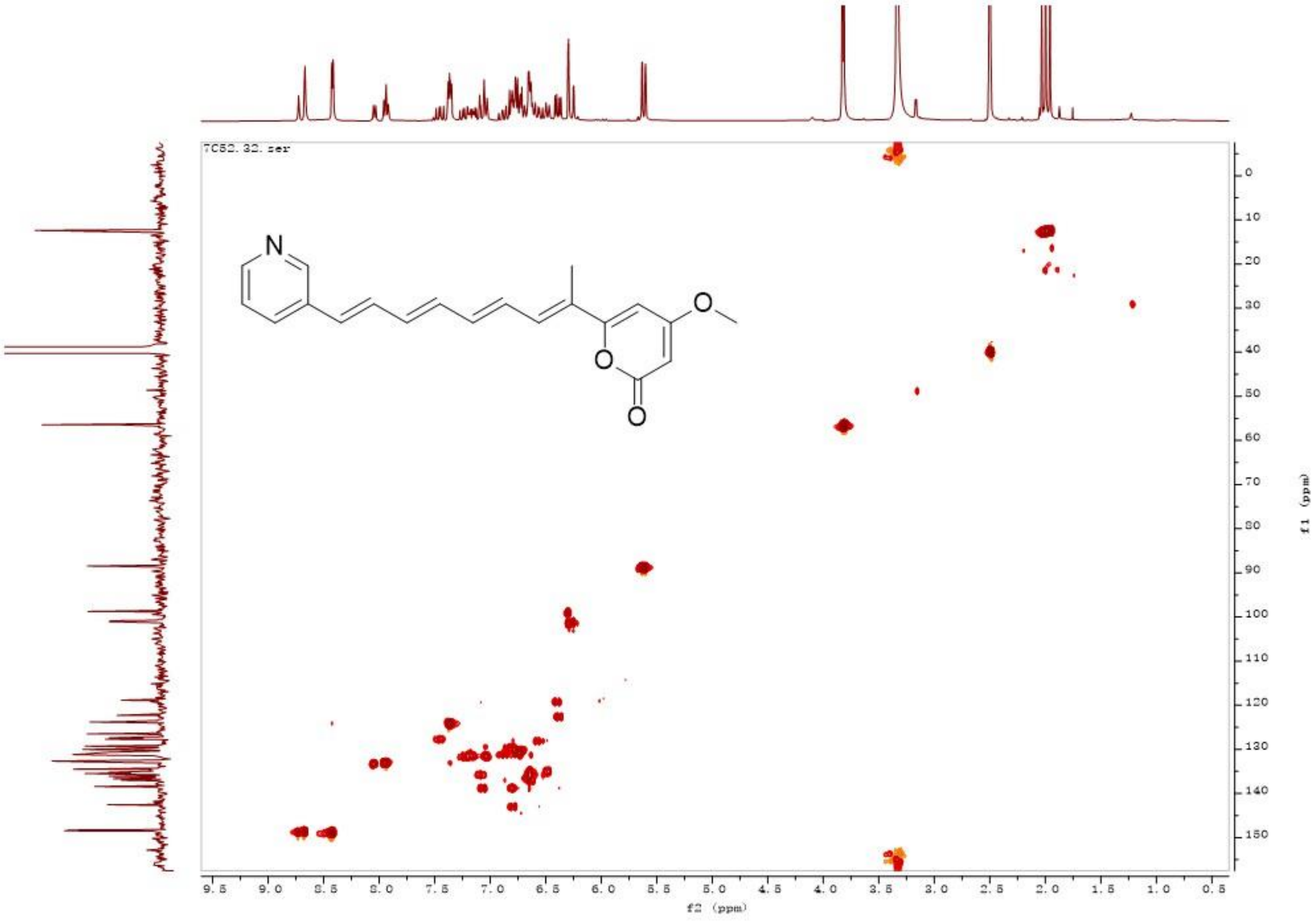

Figure S33. HMBC spectrum of pyridinopyrone H (**4**) (DMSO-d_6)

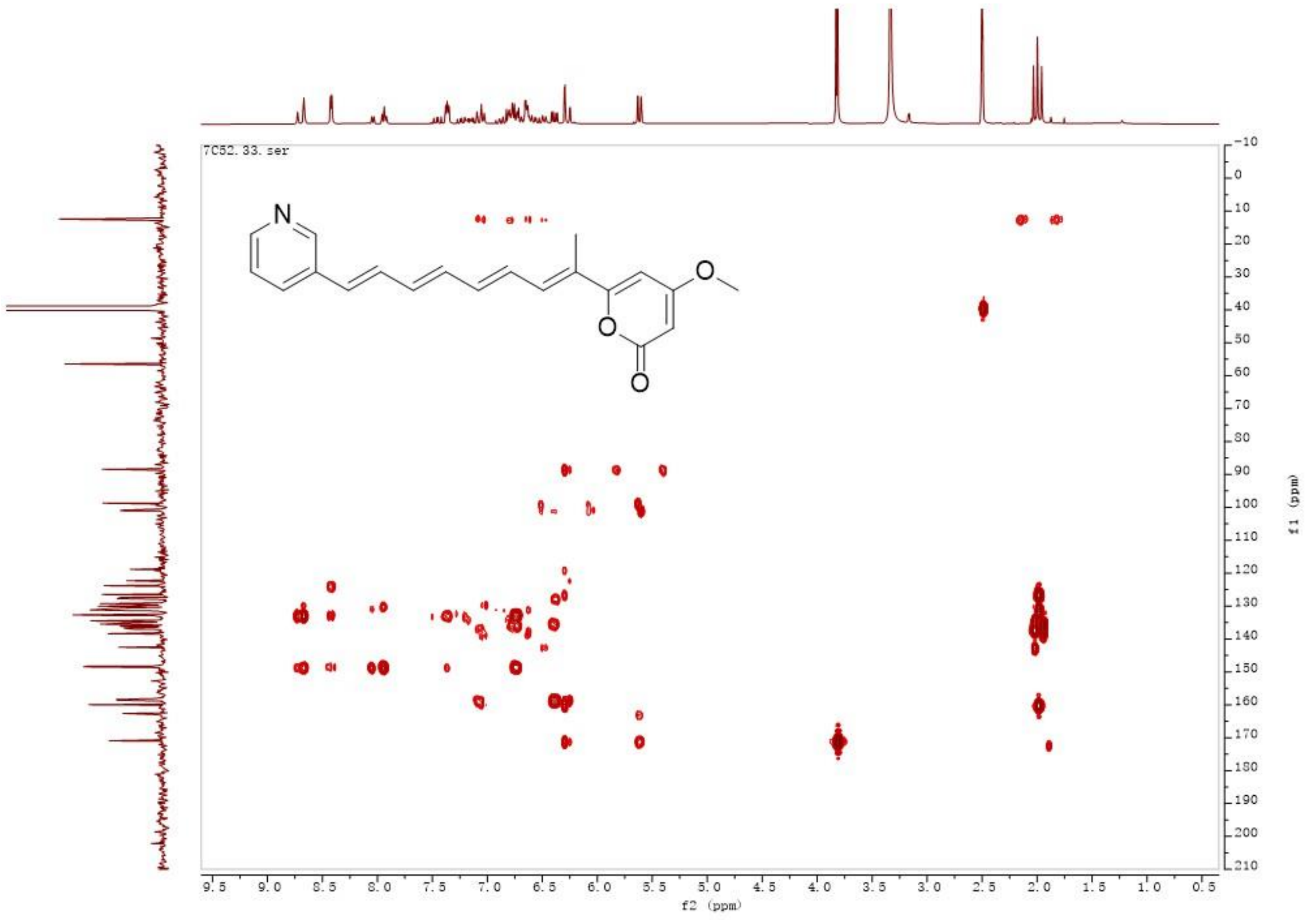

Figure S34. HRESIMS spectrum of pyridinopyrone H (**4**)

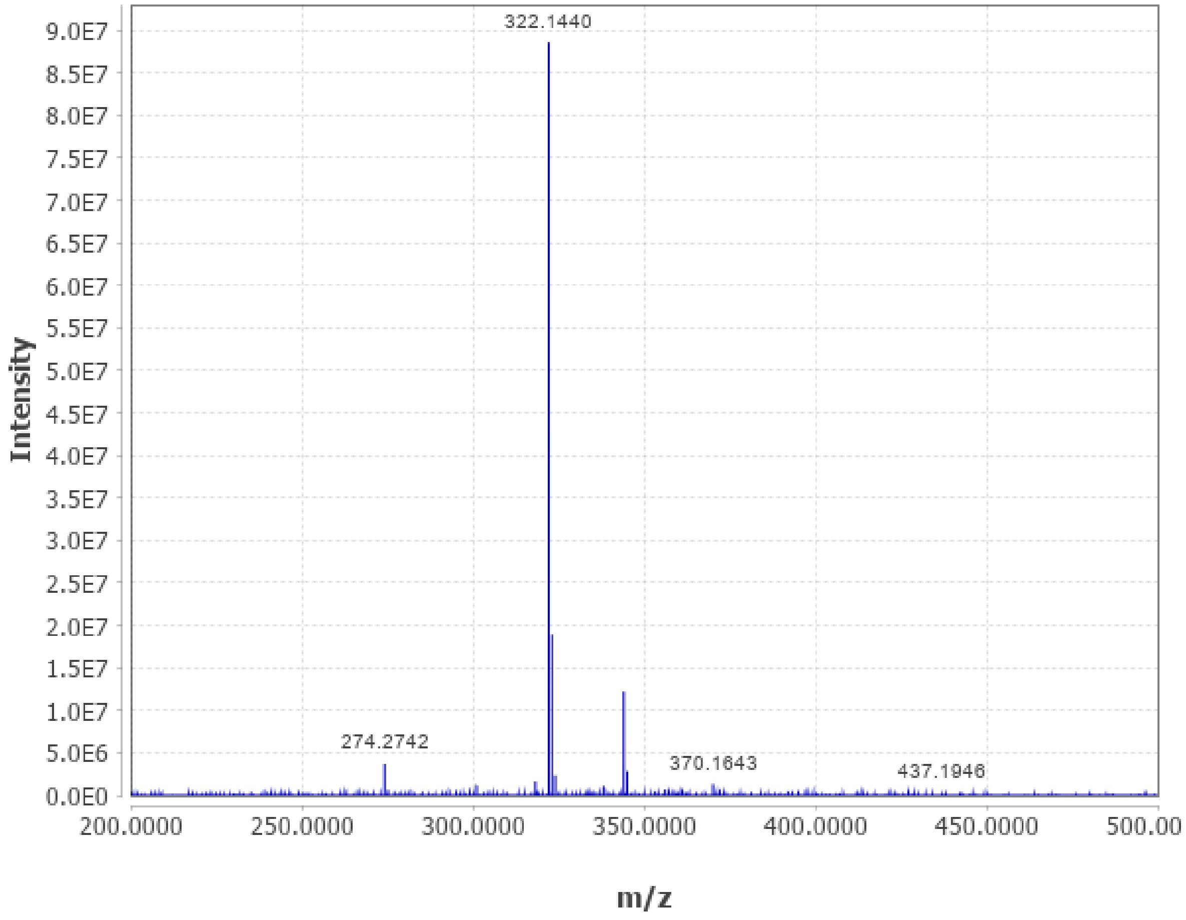

Figure S35. UV spectrum of pyridinopyrone H (**4**)

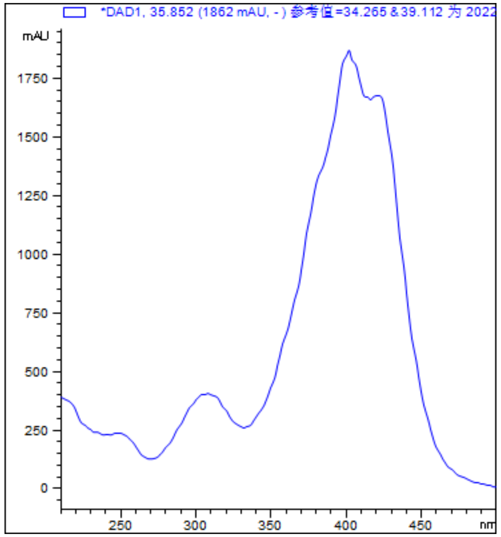

Supplement: Supplementary file 1 [file Data_Sheet_1.PDF]
